# Roles of the *Brassica napus* DELLA Protein BnaA6.RGA, in Modulating Drought Tolerance by Interacting With the ABA Signaling Component BnaA10.ABF2

**DOI:** 10.3389/fpls.2020.00577

**Published:** 2020-05-14

**Authors:** Jiajing Wu, Guanbo Yan, Zhiqiang Duan, Zhijuan Wang, Chunying Kang, Liang Guo, Kede Liu, Jinxing Tu, Jinxiong Shen, Bin Yi, Tingdong Fu, Xia Li, Chaozhi Ma, Cheng Dai

**Affiliations:** ^1^National Key Laboratory of Crop Genetic Improvement, Huazhong Agricultural University, Wuhan, China; ^2^State Key Laboratory of Agricultural Microbiology, College of Plant Science and Technology, Huazhong Agricultural University, Wuhan, China; ^3^Key Laboratory of Horticultural Plant Biology (Ministry of Education), College of Horticulture and Forestry Sciences, Huazhong Agricultural University, Wuhan, China

**Keywords:** GA, DELLA protein, *BnaRGA*, ABA, *BnaA10.ABF2*, *Brassica napus*

## Abstract

Drought is a major threat to plant growth and crop productivity. Reduced level of the gibberellin would result in increased drought tolerance, but the underlying mechanism is still unclear. In *Brassica napus*, there are four *BnaRGA* genes that code for DELLA proteins, negative regulators of GA signaling. Among them, expression of *BnaA6.RGA* was greatly induced by drought and abscisic acid (ABA). Previously, we created the gain-of-function mutant of *BnaA6.RGA*, *bnaa6.rga-D*, and the loss-of-function quadruple mutant, *bnarga* by CRISPR/Cas9, respectively. Here we show that *bnaa6.rga-D* displayed enhanced drought tolerance, and its stomatal closure was hypersensitive to ABA treatment. By contrast, *bnarga* displayed reduced drought tolerance and was less sensitive to ABA treatment, but there is no difference in drought tolerance between single *BnaRGA* mutant and WT, suggesting a functional redundancy between the *BnaRGA* genes in this process. Furthermore, we found that BnaRGAs were able to interact physically with BnaA10.ABF2, an essential transcription factor in ABA signaling. The BnaA10.ABF2-BnaA6.RGA protein complex greatly increased the expression level of the drought responsive gene *BnaC9.RAB18*. Taken together, this work highlighted the fundamental roles of DELLA proteins in drought tolerance in *B. napus*, and provide desirable germplasm for further breeding of drought tolerance in rapeseed.

## Introduction

Drought is a major stress that causes decreases in crop yield. Genetic engineering by regulating drought responsive genes are the effective approach for enhancing crop drought tolerance, which increases agricultural productivity to meet the food demand of expanding population (Zhu, 2016). Rapeseed (*Brassica napus* L., AACC, 2*n* = 38) is cultivated worldwide to produce edible oil, animal feed and biodiesel, making it an agriculturally important crop. *B. napus* is very sensitive to water deficits from germination to seed set (Zhu et al., 2016). Thus, there is a tremendous need and interest in understanding the physiology and molecular mechanism underlying of rapeseed to cope with drought stress.

The plant hormone abscisic acid (ABA) plays essential roles during drought responses (Zhu, 2002; Chaves et al., 2003; Chen et al., 2020), and its level increases under water-deficit conditions. The identification of ABA receptors PYRABACTIN RESISTANCE1 (PYR1)/PYR1-LIKE (PYL)/REGULATORY COMPONENTS OF ABA RECEPTORS (RCAR) revealed the core of the ABA-signaling pathway (Ma et al., 2009; Park et al., 2009). In the absence of ABA, subclass III SNF1-related protein kinases (SnRK2.2/2.3/2.6) are dephosphorylated through interactions with a group A protein phosphatase type 2C (PP2C) to remain inactive (Soon et al., 2012). In the presence of ABA, the PYL receptors bind with ABA and form a PYL-ABA-PP2C complex, which inhibits the phosphatase activities of PP2C (Ma et al., 2009; Park et al., 2009). As a consequence, SnRK2s are released from the SnRK2-PP2C complex and become activated through autophosphorylation. These activated SnRK2s can then phosphorylate downstream transcription factors to increase drought tolerance (Ma et al., 2009; Park et al., 2009). Among these transcription factors, the bZIP group of ABA response element (ABRE)-binding factors (ABFs) play important roles in ABA signaling transduction (Zhu, 2002). In *Arabidopsis*, there are four *ABF*s, *ABF1-4*, and their expression levels are greatly induced by drought and ABA in vegetative tissues (Fujita et al., 2005). These ABFs bind to the ABRE *cis*-elements in the promoters of downstream genes, such as *RAB18*, *RD29A*, and *RD29B* (Fujita et al., 2005). The overexpression of *ABF2* significantly increases drought tolerance in rice and tomato (Hossain et al., 2010; Hirano et al., 2012; Li et al., 2013; Zhao et al., 2016), and the constitutive expression of peanut *ABF2* in *Arabidopsis* can also enhance drought tolerance (Li et al., 2013), suggesting that *ABF2* is a master regulator of the ABA-dependent pathway.

The growth-promoting hormone gibberellin (GA) may also be involved in drought tolerance (Magome et al., 2008; Colebrook et al., 2014; Nir et al., 2014, 2017; Vishal and Kumar, 2018). GA levels are significantly reduced under drought conditions (Nelissen et al., 2018). In *Arabidopsis*, the GA deficient mutants *ga20ox1/2* and *ga3ox1/2* are more resistant to drought (Colebrook et al., 2014). Tomato plants overexpressing *GA METHYLTRANSFERASE1* (*GAMT*), which encodes a GA methyltransferase, also have enhanced tolerance levels to water-deficit stress (Nir et al., 2014). DELLA proteins are the key repressors of GA signaling (Ueguchi-Tanaka et al., 2007). In *Arabidopsis*, the DELLA protein family includes five members, REPRESSOR OF ga1-3 (RGA), GIBBERELLIC ACID INSENSITIVE and RGA-LIKE 1-3 (Hirsch and Oldroyd, 2009). Recently, a mutant of tomato *PROCERA*, which encodes a DELLA protein, displayed a rapid water loss under water-deficit conditions, while its activity increased after the removal of 17 amino acids inside the DELLA motif, reducing the water loss. In *Arabidopsis*, gain-of-function mutant *gai-1* also increases drought tolerance (Wang et al., 2020). This indicated that DELLA proteins play positive roles in drought tolerance (Nir et al., 2017; Wang et al., 2020).

DELLA proteins belong to the GRAS transcription factor family, which lack a DNA-binding domain (Yoshida et al., 2014). Thus, DELLA proteins usually form complexes with DELLA-interacting proteins (DIPs) to regulate gene expression at the transcriptional level (Van De Velde et al., 2017). A majority of the DIPs are transcription factors or transcriptional regulators. DELLA proteins can form complexes with DIPs, such as PIFs and BZR1, to prevent them from binding to the promoters of downstream genes (de Lucas et al., 2008; Bai et al., 2012; Zhang et al., 2014), or JAZ and MYC2, to prevent them from interacting with other proteins (Hou et al., 2010; Nakamura et al., 2013). In the crosstalk with ABA signaling, DELLA proteins interact with other transcription factors, such as ABI3 and ABI5, to promote the expression levels of ABA-responsive genes that inhibit seed germination (Lim et al., 2013). Thus, DELLA proteins play roles in GA-regulated biological processes or the crosstalk of GAs with other pathways through the activation or sequestration of different DIPs.

*Brassica napus* is a relatively recent allopolyploid originating from the hybridization of *Brassica rapa* (2*n* = 20, AA) with *Brassica oleracea* (2*n* = 18, CC) (Chalhoub et al., 2014). When *B. napus* is exposed to drought at the vegetative stage, both osmotic adaptive proteins, such as macromolecules, including late embryogenesis-abundant proteins, and small metabolites, including proline and trehalose, are greatly induced (Good and Zaplachinski, 1994; Dalal et al., 2009; Müller et al., 2012). Additionally, ABA rapidly accumulates in the leaves (Qaderi et al., 2006), which triggers stomatal closure (Zhu et al., 2010). Sequence analyses identified all the ABA biosynthesis and signaling components in *B. napus*, indicating that this pathway is conserved in this species (Zhu et al., 2016). Moreover, ABA pathway genes, as well as some other stress-responsive genes, are also up-regulated by drought stress in *B. napus* (Li et al., 2005; Zhu et al., 2010). The overexpression of the *B. napus ABF2* gene *BnaA10.ABF2* in *Arabidopsis* dramatically enhances drought tolerance (Zhao et al., 2016). However, the molecular basis of drought tolerance in *B. napus* is still largely unknown.

Because *B. napus* is an allotetraploid species (Chalhoub et al., 2014), it possesses 10 DELLA genes, including four homologs of *RGA*, *BnaA6.RGA*, *BnaC7.RGA*, *BnaA9.RGA*, and *BnaC9.RGA*. Previously, mutants of these *BnaRGA*s were generated using CRISPR/Cas9 technology (Yang et al., 2017). Here, we demonstrated that *BnaA6.RGA* acts as a positive regulator of drought tolerance by promoting stomatal closure through increased ABA sensitivity and subsequently by reducing water loss in response to a water deficit. Moreover, *BnaA6.RGA* regulated the expression of drought-responsive genes by directly interacting with BnaA10.ABF2, the ortholog of *Arabidopsis* ABF2. Our findings provide novel insights into the crosstalk between GA and ABA signaling pathways, and provide a useful germplasm for improving rapeseed drought tolerance.

## Results

### *BnaA6.RGA* Was Greatly Induced by Drought and Abscisic Acid

**FIGURE 1:**
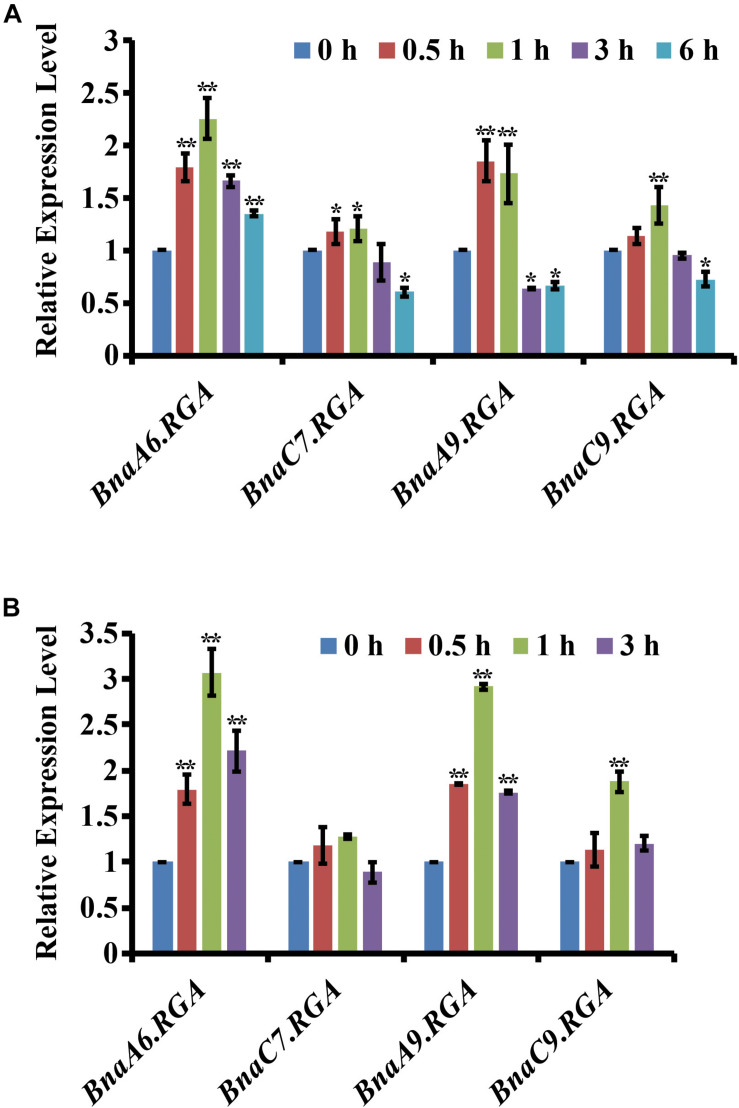
Expression of the four *BnaRGA*s in response to drought and ABA treatment. **(A)** Expression of the four *BnaRGAs* in response to drought treatment in WT analyzed by qRT-PCR. **(B)** Expression of the four *BnaRGAs* in response to ABA (100 μM) treatment in WT analyzed by qRT-PCR. These genes’ expression levels at 0 h were set as 1. *BnaGAPDH* was used as the internal control. Data are means ± SD obtained from three biological replicates. Asterisks show that the values are significantly different between the 0 h and different time point. The data were analyzed by Duncan’s multiple range tests in the ANOVA program of SPSS (*P < 0.05 and **P < 0.01).

Drought tolerance is promoted by reducing the endogenous GA level in plants (Colebrook et al., 2014). Therefore, we hypothesized that the negative regulators of GA signaling, the DELLA proteins, may also play important roles in the drought tolerance of *B. napus*. In *B. napus*, there are four *BnaRGAs* (*B. napus REPRESSOR OF ga1-3*) homologs of *Arabidopsis RGA* gene (Zhao et al., 2017). To identify *RGA* genes that are responsive to drought and ABA, we examined their expression patterns under drought or ABA conditions using quantitative RT-PCR. After the drought treatment, the expression of *BnaA6.RGA* was greatly induced at 1 h, being three times higher than in the untreated tissue, and then its level slightly decreased at 3 h (Figure 1A). Compared with *BnaA6.RGA*, the expression levels of the *BnaA9.RGA* and *BnaC9.RGA* genes were also induced by drought, but the *BnaC7.RGA* was not significant changed (Figure 1A). Similarly, the expression of *BnaA6.RGA* was also greatly induced by an exogenous application of ABA, reaching its highest level at 1 h (Figure 1B), which was almost same drought treatment. In addition, the expressions of other *BnaRGAs* were up-regulated by the ABA treatment, although to a lesser extent than that of *BnaA6.RGA* (Figure 1B). Thus, the expression of *BnaA6.RGA* is more sensitive than those of other *DELLA* genes in response to drought and ABA treatments, which suggests that *BnaA6.RGA* plays more important roles in the drought tolerance of *B. napus*.

### *BnaA6.RGA* and *BnaC7.RGA* Played Positive Roles in Drought Tolerance

Then, the genome editing of *BnaRGA*s was performed using CRISPR/Cas9 (Yang et al., 2017). Two types of mutants were generated among the transgenic plants according to the genotyping results. L4 and L6 are gain-of-function mutants of *BnaA6.RGA*, designated *bnaa6.rga-D*. In L4, there was a 6-nt deletion at the sgRNA2 target site that caused a two-amino acid deletion in the TVHYNP motif (Yang et al., 2017). In L6, there were both a 9-nt deletion at the sgRNA1 target site that caused a three-amino acid deletion in the DELLA motif and a 12-nt deletion at the sgRNA2 target site that caused a four-amino acid deletion in the TVHYNP motif (Yang et al., 2017). All the other lines possessed loss-of-function mutations. For example, there was a 118-nt deletion in L2 and 1-nt insertion in L8 at the sgRNA1 target site that caused frameshifts in *BnaA6.RGA*, designated *bnaa6.rga*. These mutations in *bnaa6.rga* also reduced the transcript level of *BnaA6.RGA* significantly (Supplementary Figure S1). These mutants provided precious materials for investigating *BnaA6.RGA*’s roles in drought tolerance in *B. napus*.

**FIGURE 2:**
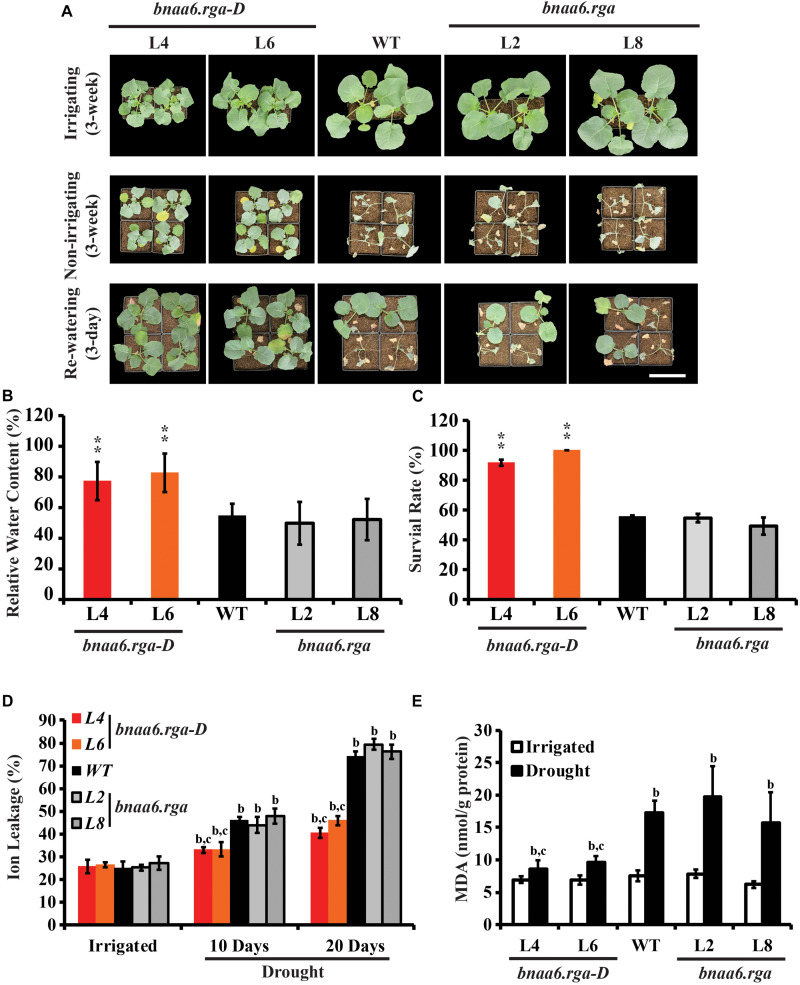
Drought tolerance phenotypes of *bnaa6.rga* and *bnaa6.rga-D*. **(A)** Images showing the phenotypes of WT, *bnaA6.rga-D*, and *bnaa6.rga* in response to progressive drought stress. Images were taken for well-watered plants, at 20 days without irrigation, and at 3 days after rehydration. **(B)** Bar graph showing the leaf relative water content of WT, *bnaA6.rga-D*, and *bnaa6.aga* under water-deficit conditions in **(A)**. **(C)** Bar graph showing the survival rates of WT, *bnaA6.rga-D*, and *bnaa6.aga* under water-deficit conditions followed by re-watering. **(D)** Bar graph showing the ion leakage of WT, *bnaA6.rga-D*, and *bnaa6.aga* in response to progressive drought stress. **(E)** Bar graph showing the MDA content of WT, *bnaA6.rga-D*, and *bnaa6.aga* before and after drought treatment. In **(B)** and **(C)** data are means ± SD (*n* = 10–15) obtained from three biological experiments. Asterisks show that the values are significantly different between the WT and different mutants at the same time point. The data were analyzed by Duncan’s multiple range tests in the ANOVA program of SPSS (*P < 0.05 and **P < 0.01). In **(D)** and **(E)** letters indicate statistically significant differences between b: drought treatment vs control WT and c: drought treatment of mutants vs drought treatment of WT at *P* < 0.05 (Duncan’s multiple range tests). In **(A–E)** L4 and L6: two individual *bnaa6.rga-D* lines; L2 and L8: two individual *bnaa6.rga* lines; WT: *Westar*.

To determine whether *BnaA6.RGA* regulates drought resistance, 3-week-old wild type (WT; Westar), and *bnaa6.rga* and *bnaa6.rga-D* mutants, grown in pots were subjected to drought stress by withholding water for 20 days and then re-watering the plants for 3 days. After 20 days of water deprivation, the relative soil water content was almost the same in each pot (Supplementary Figure S2). We found almost half of the WT plants wilted, but the *bnaa6.rga-D* plants remained turgid (Figure 2A). The leaf relative water content (RWC) was consistent with the drought phenotype, being 77.3–82.7% in *bnaa6.rga-D* plants, which was greater than in WT (54.6%) (Figure 2B). After 3 days of re-watering, *bnaa6.rga-D* plants recovered well, and the survival rates of the two *bnaa6.rga-D* lines reached 91.7 and 100%. In contrast, the WT survival rate was only 55.6% (Figure 2C), which suggested that *bnaa6.rga-D* is more tolerant to drought stress. However, the survival rate and leaf RWC of *bnaa6.rga* were not significantly different from those of WT (Figures 2A–C).

Ion leakage is an important indicator of cell injury. Then, the ion leakage was measured in irrigated versus water-deprived WT, *bnaa6.rga-D* and *bnaa6.rga* leaves. Under irrigated condition, no significant differences were found between the different lines (Figure 2D). After 10 days without irrigation, the ratio of ion leakage of *bnaa6.rga-D* was about 33.9%, which was less than WT (46.2%) (Figure 2D). After 20 days, the differences of ratio of ion leakage between *bnaa6.rga-D* and WT were much greater (Figure 2D), suggesting that membrane damage of WT was more serious than *bnaa6.rga-D*. Meanwhile, at the same drought condition, the ion leakage of *bnaa6.rga* was no different from WT (Figure 2D). Then, malondialdehyde (MDA) was measured in WT, *bnaa6.rga-D* and *bnaa6.rga* leaves, before or after the drought treatment. Compared to the WT, the MDA was less accumulated in *bnaa6.rga-D*, following exposure to the same drought condition (Figure 2E). These results indicated that *bnaa6.rga-D* could enhance the drought tolerance in *B. napus*.

Previously, the gain-of-function mutants of *BnaC7.RGA*, *ds-3*, was obtained by screening rapeseed EMS library (Zhao et al., 2017). A substitution of proline to leucine was identified in *ds-3* in the conserved VHYNP motif, which is essential for GA-dependent interaction between GA receptor GID1 and DELLA proteins (Zhao et al., 2017). Then, we also tested the *ds-3* in response to drought. *ds-3* and *WT* (Huashang5, *HS5*) were subjected to the same drought treatment. Like *bnaa6.rga-D*, *ds-3* plants remained turgid (Supplementary Figure S3A) after 3-week without watering, and the leaf RWC was about 95% (Supplementary Figure S3B). After 3 days of re-watering, *ds-3* plants recovered well, and the survival rates was about 100% (Supplementary Figure S3C). The ion leakage and MDA content of *ds-3* were less than WT (Supplementary Figures S3D,E). In contrast, the *HS5* survival rate was almost 0% (Supplementary Figure S3C), which suggested that *ds-3* is more tolerant to drought stress. Although *BnaC7.RGA* was not significant induced by drought or ABA, the gain-of-function mutants of *BnaC7.RGA* also showed strong drought tolerance, indicating that RGA proteins play the same functions in rapeseed drought tolerance.

### *BnaRGA*s Played Redundant Roles in Drought Tolerance

**FIGURE 3:**
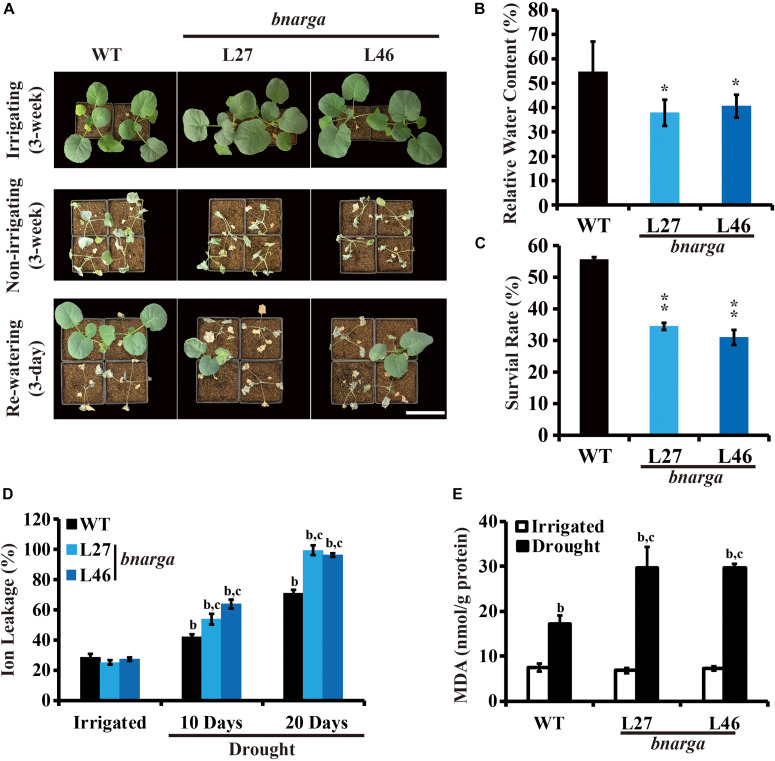
Drought tolerance phenotypes of *bnarga*. **(A)** Images showing the phenotypes of WT, and *bnarga* in response to progressive drought stress. Images were taken for well-watered plants, at 20 days without irrigation, and at 3 days after rehydration. **(B)** Bar graph showing the leaf relative water content of WT, *bnaaga* under water-deficit conditions in **(A)**. **(C)** Bar graph showing the survival rates of WT and *bnarga* under water-deficit conditions followed by re-watering. **(D)** Bar graph showing the ion leakage of WT, and *bnaaga* in response to progressive drought stress. **(E)** Bar graph showing the MDA content of WT, and *bnaaga* before and after drought treatment. In **(B)** and **(C)** data are means ± SD (*n* = 10–15) obtained from three biological experiments. Asterisks show that the values are significantly different between the WT and different mutants at the same time point. The data were analyzed by Duncan’s multiple range tests in the ANOVA program of SPSS (*P < 0.05; **P < 0.01). In **(D)** and **(E)** letters indicate statistically significant differences between b: drought treatment vs control WT and c: drought treatment of mutants vs drought treatment of WT at *P* < 0.05 (Duncan’s multiple range tests). In **(A–E)** L27 and L46: two individual *bnarga* lines; WT: *Westar*.

Because there are four *BnaRGA*s, we hypothesized that the other *BnaRGA* genes may act redundantly during drought tolerance. To test this hypothesis, single mutants of the other *BnaRGA*s (*bnac7.rga*, L7; *bnaa9.rga*, L5 and L16; and *bnac9.rga*, L3) and the quadruple mutant *bnarga*, L27 and L46 (Yang et al., 2017), were subjected to the same drought treatment. Like *bnaa6.rga*, the survival rates were similar among the single mutants and WT (Supplementary Figure S4). In contrast, a majority of the *bnarga* plants wilted after three weeks without watering (Figure 3A). The leaf RWCs of *bnarga* plants ranged from 37.9 to 40.6% (Figure 3B), which was lower range than that of the WT. After re-watering, the two lines of *bnarga* plants, L27 and L46, recovered poorly, with survival rates of only 34.4 and 31.0%, respectively, indicating that *bnarga* is more sensitive to drought stress than WT (Figure 3C). After 10 days without irrigation, the ratio of ion leakage of *bnarga* was about 55–60%, which was more than WT (46.2%) (Figure 3D). After 20 days, the differences of ratio of ion leakage between *bnarga* and WT were much greater (Figure 3D), suggesting that membrane damage of *bnarga* was more serious than WT. The MDA was more accumulated in *bnarga*, following exposure to the same drought condition (Figure 3E). Thus, *BnaA6.RGA* and *BnaC7.RGA* positively regulates drought tolerance in *B. napus*, while other *BnaRGA*s have redundant functions in this process.

### *BnaRGA* Promoted Stomatal Closure in Response to Abscisic Acid

**FIGURE 4:**
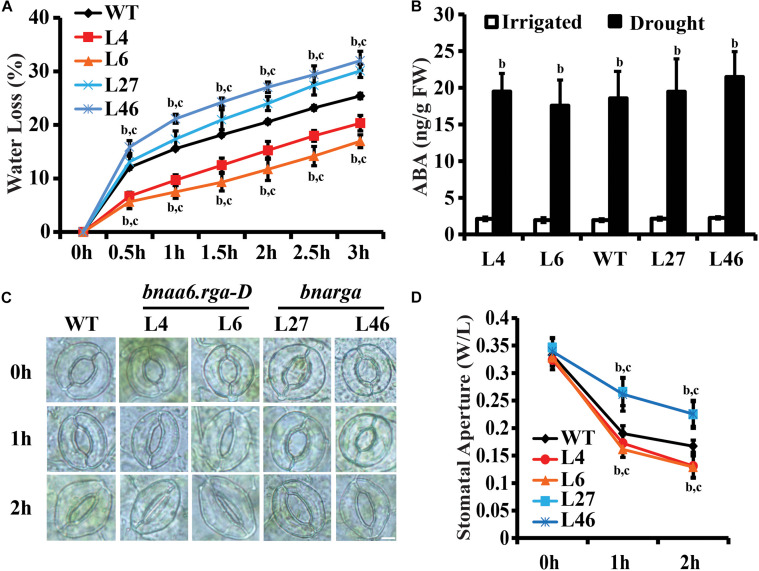
Water loss of detached leaves and stomatal aperture after ABA treatment of *bnaa6.rga-D* and *bnarga*. **(A)** Water loss of detached leaves of *bnaa6.rga-D*, *bnarga*, and WT. Leaves at similar developmental stages were excised and weighed at the indicated time after detachment. The proportion of fresh weight losses was calculated on the basis of the initial weight of the leaves. **(B)** Bar graph showing the ABA content of WT, *bnaA6.rga-D*, and *bnaaga* in response to progressive drought stress. **(C)** Images of the representative stomata of *bnaa6.rga-D*, *bnarga*, and WT at 0, 1, and 2 h with or without ABA (1 μM) treatment. Scale bars: 10 μm. **(D)** Line graph showing the stomatal apertures measured from **(C)**. In **(A)**, **(B)**, and **(D)** data are means ± SD [*n* = 5–6 for **(A)** and **(B)**; *n* = 150–200 for **(D)**] obtained from three biological experiments. In **(A)**, **(B)**, and **(D)** letters indicate statistically significant differences between b: drought (or ABA) treatment vs control WT and c: drought (or ABA) treatment of mutants vs drought (or ABA) treatment of WT at *P* < 0.05 (Duncan’s multiple range tests). In **(A–D)** L4 and L6: two individual *bnaa6.rga-D* lines; L2 and L8: two individual *bnaa6.rga* lines; L27 and L46: two individual *bnarga* lines; WT: *Westar*.

Reducing water loss is a key determinant of drought tolerance (Xiong et al., 2002). The *BnaRGA* mutants exhibited different sensitivities to drought stress. To uncover the underlying causes, weights of the detached leaves from these mutants were measured every half hour for 3 h after detachment. The *bnarga* quadruple mutants displayed significantly greater water loss rate, while *bnaa6.rga-D* and *ds-3* showed a significantly lower water loss rate in comparison to that of WT (Figure 4A, Supplementary Figure S5A). Thus, the expression of *BnaA6.RGA* and *BnaC7.RGA* appears to reduce drought-induced water loss.

The drought stress induces ABA biosynthesis (McAdam and Brodribb, 2016), which, in turn, promotes stomatal closure. We first analyzed ABA levels in well-watered versus water-deprived WT, *bnaa6.rga-D*, and *bnarga* leaves. After 10 days without irrigation, the soil RWC of all plants reached round 40%. Under irrigation, no significant differences in ABA content were found between the different lines (Figure 4B). While the water deficit treatment increased ABA levels in all lines, which was no significant differences in different lines (Figure 4B). These results suggest that BnaRGAs does not promote ABA accumulation in *B. napus* leaves.

Measuring ABA-induced stomatal closure is a well-established assay for studying plant responses to drought stress (Mustilli et al., 2002). To evaluate whether BnaRGAs affects stomatal response to ABA, we treated peeled abaxial epidermal strips taken from *bnaa6.rga-D*, *ds-3*, *bnarga*, and their relative wildtype leaves with ABA treatment and monitored stomatal closure. The stomata of WT (Westar), *bnaa6.rga-D* and *bnarga* were induced to be wide open before the ABA treatment (Figures 4C,D). After the ABA treatment, the stomatal apertures of all these plants decreased; however, *bnaa6.rga-D* was more sensitive than WT, while the *bnarga* plants were less sensitive (Figures 4C,D). Specifically, 1 h after the ABA treatment, the average stomatal aperture (width/length) of the WT was 0.19, while those of the two *bnaa6.rga-D* were 0.16 to 0.17, and that of *bnarga* was 0.26 (Figures 3C,D, *P* < 0.01). At 2 h after the ABA treatment, the differences between stomatal apertures were much greater (Figures 4C,D). The same pattern was found between *ds-3* and *HS5* (Supplementary Figures S5A–C). Thus, the stomatal closures of the *BnaRGA* mutants in response to the ABA treatment were consistent with the wilting phenotypes under drought-stress conditions.

### BnaRGAs Physically Interacted With BnaA10.ABF2

**FIGURE 5:**
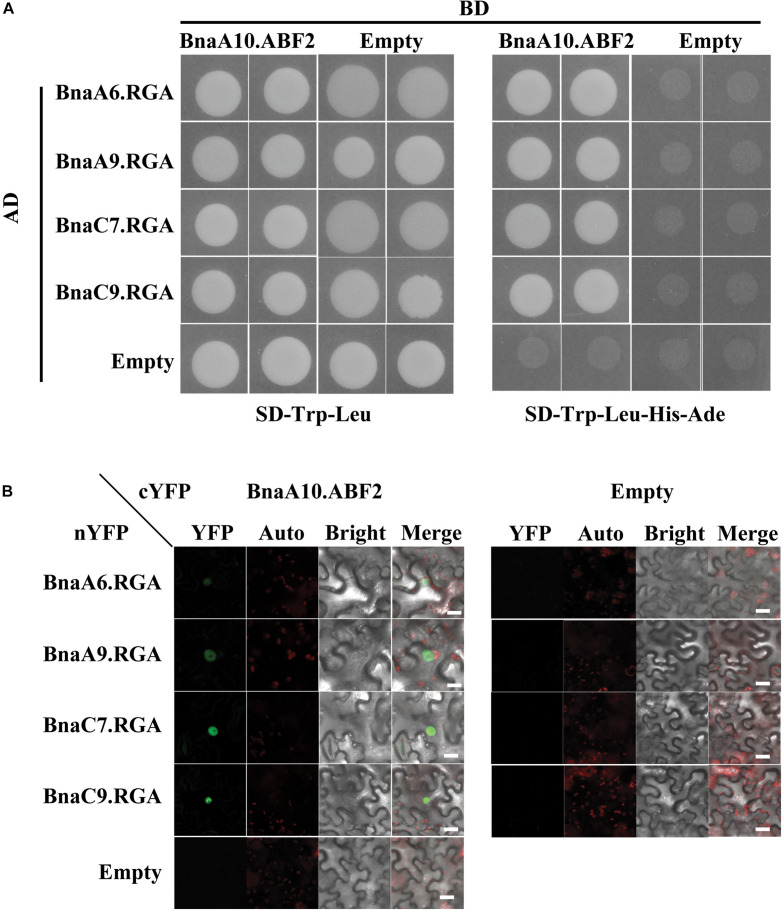
Each of the four BnaRGAs physically interact with BnaA10.ABF2. **(A)** Each of the four BnaRGAs interacted with BnaA10.ABF2 in the Y2H assay. **(B)** Each of the four BnaRGAs interacted with BnaA10.ABF2 in the BiFC assay performed in the *N. benthamiana* leaves. Scale bars: 50 μm. Images were acquired by confocal microscope using the identical settings. YFP, yellow fluorescent protein; auto, chloroplast auto fluorescence; bright, bright field; merge, the figure merged by YFP, auto, and bright.

DELLA proteins usually play roles by interacting with other transcription factors (Van De Velde et al., 2017). In *Arabidopsis*, RGA can interact with the bZIP transcription factor ABI5 to regulate seed germination (Lim et al., 2013). The bZIP transcription factor *BnaA10.ABF2* positively regulates plant drought tolerance when transformed into *Arabidopsis* (Zhao et al., 2016). Thus, we hypothesized that BnaA6.RGA might directly interact with BnaA10.ABF2. We used a yeast two-hybrid system to test this possibility, and it indicated that these two proteins interact (Figure 5A). A phylogenetic analysis revealed that the BnaA10.ABF2 protein belongs to the ABF2 clade of the ABRE family along with three other paralogs (Supplementary Figure S6). Three other BnaRGA proteins could also physically interact with BnaA10.ABF2 (Figure 5A). Then, bimolecular fluorescence complementation (BiFC) was performed to examine the interactions between BnaA10.ABF2 and the four BnaRGA proteins. The YFP fluorescence occurred specifically in the nucleus only when *BnaA10.ABF2-cYFP* and each *BnaRGA-nYFP* were expressed simultaneously in tobacco leaves (Figure 5B). Without the GRAS domain, BnaA6.RGA and BnaC7.RGA could not interact with BnaA10.ABF2 (Supplementary Figure S7). These results suggest that BnaA10.ABF2 physically interacted with BnaRGAs to form a protein complex.

### BnaRGA-BnaA10.ABF2 Complex Enhances the Expression of *BnaC9.RAB18*

**FIGURE 6:**
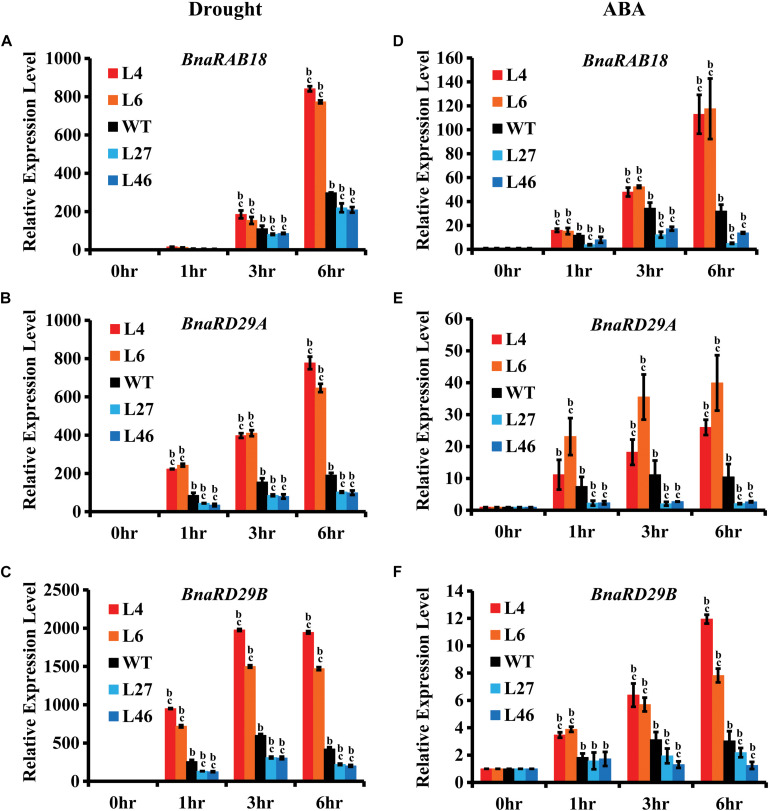
Expression of *BnaRAB18*, *BnaRD29A*, and *BnaRD29B* in *bnaa6.rga-D* and *bnarga* in response to drought and ABA treatment. **(A–C)** Expression levels of *BnaRAB18* **(A)**, *BnaRD29A* **(B)**, and *BnaRD29B* **(C)** in *bnaa6.rga-D*, *bnarga*, and WT after drought treatment examined by qRT-PCR. **(D–F)** Expression levels of *BnaRAB18* **(D)**, *BnaRD29A* **(E)**, and *BnaRD29B* **(F)** in *bnaa6.rga-D*, *bnarga*, and WT after ABA treatment examined by qRT-PCR. The expressions level of each gene at 0 h was set as 1. *BnaGAPDH* was used as the internal control. Data are means ± SD obtained from three biological replicates. In **(A–F)** letters indicate statistically significant differences between b: drought (or ABA) treatment vs control WT and c: drought (or ABA) treatment of mutants vs drought (or ABA) treatment of WT at *P* < 0.05 (Duncan’s multiple range tests). In **(A–F)** L4 and L6: two individual *bnaa6.rga-D* lines; L2 and L8: two individual *bnaa6.rga* lines; L27 and L46: two individual *bnarga* lines; WT: *Westar*.

Because BnaRGA proteins physically interact with BnaA10.ABF2, we speculated that BnaRGAs may affect the expression of *ABF2*’s downstream genes. To investigate this possibility, the expression levels of its downstream genes were examined in *bnaA6.rga-D*, *bnarga* and WT after the drought or ABA treatment. After the drought treatment, the expression of *BnaRAB18* was about 12.8–841.8 times induced in *bnaa6.rga-D* (Figure 6A), which was much greater than in WT. Under the same conditions, the expression of *BnaRAB18* was induced to a lesser extent in *bnarga* (Figure 6A). Similarly, *BnaRD29A* and *BnaRD29B* were also significantly up-regulated in *bnaa6.rga-D* and up-regulated to a lesser extent in *bnarga* compared with WT (Figures 6B,C). After the ABA treatment, these three downstream genes were all greatly induced in *bnaa6.rga-D*, but were induced to a significantly lesser extent in *bnarga* (Figures 6D–F). The same results were observed in *ds-3* after drought treatment (Supplementary Figures S8A–C). Thus, *BnaA6.RGA* and *BnaC7.RGA* may promote drought tolerance by enhancing the expression of *BnaA10.ABF2*’s downstream genes.

**FIGURE 7:**
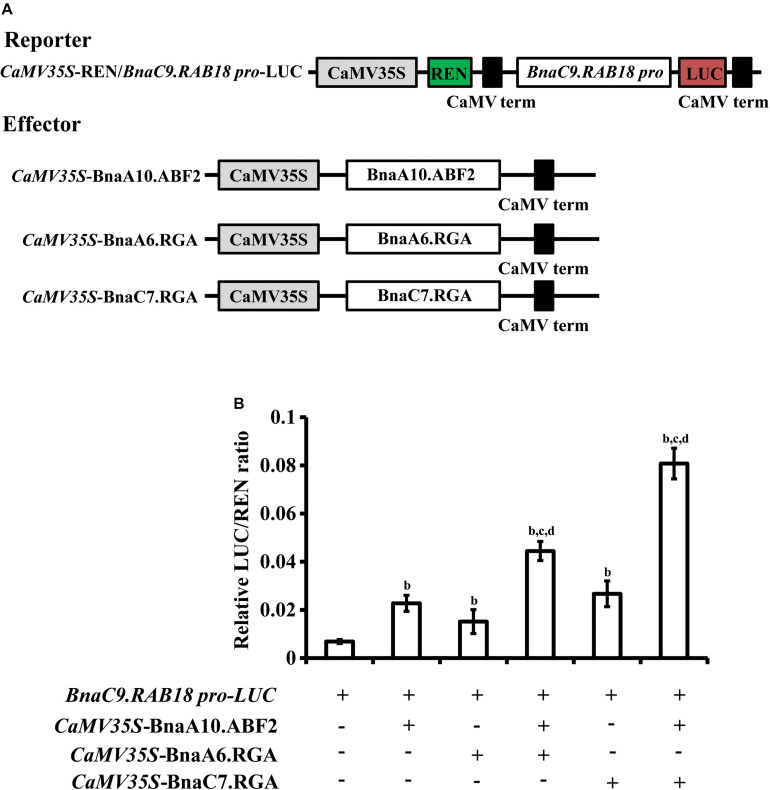
Transcriptional regulation of *BnaC9.RAB18* by BnaA10.ABF2 was enhanced by BnaA6.RGA and BnaC7.RGA, respectively. **(A)** Schematic representation of the constructs used for the dual-luciferase assay. The reporter construct contains the firefly luciferase driven by *BnaC9.RAB18* promoter, and the Renilla luciferase (REN) driven by the *CaMV 35S promoter*. The effector constructs contain BnaA6.RGA, BnaC7.RGA, and BnaA10.ABF2 driven by the *CaMV 35S* promoter, respectively. **(B)** Bar graph showing the LUC/REN ratios in the dual-luciferase assay. In **(B)** letters indicate statistically significant differences between b: co-infiltrated effectors with reporters (*BnaC9.RAB18p*-LUC) vs reporters only, c: co-infiltrated effectors (BnaA6.RGA + BnaA10.ABF2, or BnaC7.RGA + BnaA10.ABF2) with reporters vs co-infiltrated effectors (BnaA10.ABF2) with reporters, and d: co-infiltrated effectors (BnaA6.RGA + BnaA10.ABF2, or BnaC7.RGA + BnaA10.ABF2) with reporters vs co-infiltrated effectors (BnaA6.RGA, or BnaC7.RGA) with reporters at *P* < 0.05 (Duncan’s multiple range tests).

In *Arabidopsis*, ABF2 activates expression of *RAB18* (Fujita et al., 2005). Therefore, we used the dual luciferase reporter assay to determine whether BnaA10.ABF2 could promote the expression of *BnaRAB18* in *B. napus*. The reporter vector contained a firefly luciferase gene driven by the *BnaC9.RAB18* promoter and a renilla luciferase (REN) gene driven by the *CaMV 35S* promoter. *BnaA6.RGA*, *BnaC7.RGA* and *BnaA10.ABF2* were each driven by the *CaMV 35S* promoter in independent effector vectors (Figure 7A). The LUC activity was measured for different combinations. Compared with the expression of *pBnaC9.RAB18-LUC* only, the LUC enzyme’s activity level was 3.7 times greater when *BnaA10.ABF2* was co-expressed (Figure 7B). Furthermore, when *BnaA6.RGA* or *BnaC7.RGA* was expressed together with *BnaA10.ABF2*, the activity of *pBnaC9.RAB18-LUC* was increased by 6.5 or 11.8 times, respectively (Figure 7B). Without the GRAS domain, BnaA6.RGA and BnaC7.RGA could not interact with BnaA10.ABF2, and the expression levels of the reporter genes were less enhanced (Supplementary Figure S9). Collectively, these results suggested that BnaA6.RGA and BnaC7.RGA may form a complex with BnaA10.ABF2 to promote the expression of *BnaC9.RAB18*.

In *Arabidopsis*, the RGA protein promotes the expression of *XERICO*, an E3 ligase, which promotes destabilization of the ABA catabolic gene to antagonize GA effects (Zentella et al., 2007). After the drought treatment, the expression of *BnaXERICO* was 6.1 to 18.2 times induced in *bnaa6.rga-D* (Supplementary Figure S10A), which was much greater than in WT. Under the same conditions, the expression of *BnaXERICO* was not significantly induced in *bnarga* (Supplementary Figure S10A). Similarly, *BnaA10.ABF2* and *BnaC6.ABF2* (a paralog of *BnaABF2*) were also significantly up-regulated in *bnaa6.rga-D* and up-regulated to a lesser extent in *bnarga* (Supplementary Figure S10B). Thus, DELLA proteins appear to positively regulate ABA metabolic- and signaling-related genes.

### The Expression Levels of GA Signaling Genes Were Suppressed by Drought

In plants, DELLA proteins are degraded by GIBBERELLIN INSENSITIVE DWARF1 (GID1)-mediated pathways (Ueguchi-Tanaka et al., 2007). Therefore, we speculated that these GA signaling genes would be responsive to drought. To investigate this possibility, the expression levels of GA signaling genes were examined in *bnaA6.rga-D*, *bnarga*, and WT after the drought treatment. At 1 h after the drought treatment, the expression levels of *BnaGID1a*, and *BnaGID1c*, and *BnaSLY1* were greatly reduced, and then increased (Supplementary Figure S11), suggesting that GA negatively regulates plant drought tolerance by decreasing the stabilities of DELLA proteins. The expression of *BnaGID1a* was about 1.5–6.9 times induced in *bnaa6.rga-D* after drought treatment (Supplementary Figure S11A), which was much greater than in WT. Under the same conditions, the expression of *BnaGID1a* was repressed in *bnarga* (Supplementary Figure 11A). Similarly, *BnaGID1b* were also significantly up-regulated in *bnaa6.rga-D* and suppressed in *bnarga* compared with WT (Supplementary Figure S11B). However, the expression pattern of *BnaSLY1* was no difference among *bnaA6.rga-D*, *bnarga*, and WT after the drought treatment (Supplementary Figure S11C). The expression levels of *BnaGID1a* and *BnaGID1c* were suppressed, and then increased, indicating that the feedback regulation between *DELLA* and *GID1* could promote the latter’s transcription.

## Discussion

### *BnaA6.RGA* Plays Important Roles in Controlling the Drought Tolerance of *B. napus*

Under water-deficit conditions, ABA and other small molecules rapidly accumulate, which confers drought tolerance in *B. napus* (Zhu et al., 2010). Sequence analyses have identified all the ABA biosynthesis and signaling components in *B. napus*, indicating that this pathway is conserved in this species (Zhu et al., 2016). Although ABA pathway genes, as well as some other stress responsive genes, are up-regulated by drought stress in *B. napus* (Li et al., 2005; Zhu et al., 2010), whether these genes play important roles during drought tolerance remains obscure owing to the lack of genetic evidences. Here, our results indicated that BnaRGA proteins, key repressors of GA signaling, promoted drought tolerance by interacting with BnaABF2 in *B. napus*. The identification of these GA genes provided us insights into the regulatory mechanisms of drought resistance in *B. napus*.

### The Gibberellin Pathway Mediates the Regulation of Drought Tolerance

Gibberellin mediates various developmental processes throughout the life cycle of the plant, and a GA deficiency results in severe dwarfism (Ueguchi-Tanaka et al., 2007). In addition to promoting plant growth and development, GA biosynthesis or signaling is involved in modulating plant abiotic resistance to stresses, such as drought, salinity, and other environmental stimuli (Magome et al., 2008; Colebrook et al., 2014; Nir et al., 2014, 2017). Specifically, the GA deficient mutants *ga20ox1/2* and *ga3ox1/2* are more resistant to drought in *Arabidopsis* (Colebrook et al., 2014). Overexpressing the *GAMT* gene, which encodes a GA methyltransferase, enhances tolerance to water-deficit stress in tomato (Nir et al., 2014). The loss of *DELLA* encoding genes, such as the mutant *procera* in tomato (Nir et al., 2017), results in rapid water loss under water-deficit conditions, and the daily transpiration level is decreased in the tomato *GID1* double mutant (Illouz-Eliaz et al., 2019). Although GA is critical for stress responses in *Arabidopsis* and tomato, there are no reports on its roles in *B. napus*. We found that gain-of-function of *BnaA6.RGA* and *BnaC7.RGA* mutants (*bnaa6.rga-D* and *ds-3*) were more tolerant to drought. However, the loss of the four *BnaRGA* genes (*bnarga*) led to a hypersensitivity to drought (Figure 3), suggesting that the functions of GA pathway genes in plant drought tolerance are conserved.

DELLA family proteins are key repressors in GA signal transduction (Van De Velde et al., 2017). When the GA level is increased, DELLA proteins interact with GID1 and are degraded by the ubiquitin-dependent proteasome pathway to promote the expression of GA-responsive genes (Ueguchi-Tanaka et al., 2007). The expression levels of the positive regulators of GA signaling, such as GA receptor (*GID1a/c*) and F-box (*SCF^SLY1/2^*) genes, were greatly reduced by drought treatments, consistent with the results in *Arabidopsis* (Kilian et al., 2007), which might stimulate DELLA protein accumulation. However, expression levels of GID1 were first reduced and then increased, which was depended on *BnaRGA* (Supplementary Figure S11), indicating that the feedback regulation between *DELLA* and *GID1* could promote the latter’s transcription, as reported in *Arabidopsis* (Zentella et al., 2007). Therefore, we speculate that GA negatively regulates plant drought tolerance by suppressing the stability levels of DELLA proteins.

As an allotetraploid species, a majority of genes in *B. napus* are present in multiple copies that share high sequence similarities. *BnaA6.RGA*, *BnaA9.RGA*, and *BnaC9.RGA* were induced by drought and ABA (Figure 1), suggesting that other DELLA proteins regulate drought responses together with *BnaA6.RGA*. Consistent with this hypothesis, no significant differences were found between the single mutants of the four *BnaRGA*s and WT after the drought treatment, while the quadruple mutant *bnarga* significantly decreased the sensitivity of stomatal closure. These findings indicate that RGA proteins function redundantly in enhancing drought tolerance in *B. napus*. Although the expression pattern was quite different among the four paralogs of BnaRGA after the drought treatment, protein interaction assay indicated that all four BnaRGAs could interact with BnaA10.ABF2, and *ds-3* also displayed more drought tolerance than WT, suggesting different BnaRGA-BnaA10.ABF2 complex might co-activate drought related genes in different tissues or at the different time point.

### BnaRGA Directly Interacted With the Abscisic Acid-Signaling Component BnaABF2 to Regulate Drought Tolerance

Abscisic acid plays important roles in drought tolerance. ABA-mediated stomatal closure is involved in drought tolerance (Xiong et al., 2002). The ABA-induced stomatal closure rate was accelerated in *bnaa6.rga-D*, suggesting that ABA’s response was enhanced. However, the effects of DELLA on stomatal aperture are strongly suppressed in ABA-deficient mutants (Nir et al., 2017), indicating that these functions are ABA-dependent. DELLA proteins belong to a subgroup of the GRAS transcription factor family, which lack a DNA-binding domain (Yoshida et al., 2014). Thus, DELLA proteins usually function by interacting with other DNA-binding proteins to regulate the transcriptional activity of downstream genes (Van De Velde et al., 2017). In this way, DELLA proteins not only promote ABA biosynthesis (Zentella et al., 2007; Piskurewicz et al., 2008) but also the signaling components (Lim et al., 2013). Here, BnaA10.ABF2 interacted directly with each of the four BnaRGAs (Figure 4), providing a direct link between the ABA and GA signaling pathways during drought tolerance. Moreover, this protein interaction enhanced the expression of downstream drought-responsive genes (Figure 7). These results revealed a regulatory mechanism underlying drought tolerance in *B. napus*.

In *Arabidopsis*, the RGA protein promotes the expression of *XERICO* which destabilized the ABA catabolic genes (Zentella et al., 2007). Similarly, we found that the expression of *BnaXERICO* was up-regulated in *bnaa6.rga-D* and down-regulated in *bnarga* (Supplementary Figure S10A). Additionally, the ABA signaling genes *BnaA10.ABF2* and *BnaC6.ABF2* were also induced in *bnaa6.rga-D* (Supplementary Figure S10B). These results suggested that DELLA proteins positively regulate stomatal movement by up-regulating ABA signaling-related genes and down-regulating ABA catabolic genes. After the long-term drought treatment, drought induces similar levels of ABA accumulation in *bnaa6.rga-D* and *bnarga* lines, indicating that drought induced ABA accumulation might include BnaRGA-dependent and -independent way.

The *N*-terminal DELLA domain is important for the degradation of these proteins. Plants constitutively expressing a truncated DELLA, lacking the DELLA or TVHYNP motif, have growth defects that mimic a GA shortage, such as severe dwarfism, which is significantly reduced the crop yield (Fleet and Sun, 2005; Ueguchi-Tanaka et al., 2007; Zhao et al., 2017). There are several strategies have been developed for generating transgenic plants which is improved one agronomic trait without affecting others. For example, expression levels of *PRO* driven by guard cell-specific promoters greatly increase tomato drought tolerance but have no obvious effects on plant growth (Nir et al., 2017). The other strategy uses CRISPR/Cas9 genome editing toolkits to generate diverse *cis*-regulatory alleles that provide beneficial quantitative variation for breeding (Rodriguez-Leal et al., 2017). These reports provide new strategies to generate drought-tolerant crops and speed up molecular breeding in the future.

**FIGURE 8:**
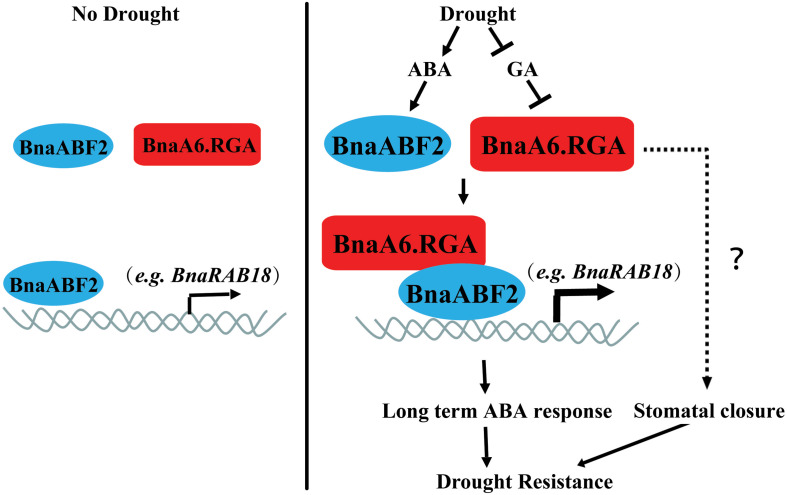
Working model for the regulatory mechanism of *BnaA6.RGA* in the drought stress responses. Drought leads to an ABA accumulation and a GA reduction, which induces the expression of ABF2 and promotes the RGA accumulation. The BnaA6.RGA-BnaABF2 complex positively regulate drought responsive genes (such as *BnaRAB18*) expression levels, resulting in enhanced ABA signaling, which then increases the plant’s drought resistance. In addition, the accumulated BnaA6.RGA might enhance guard cell sensitivity to ABA by an unknown mechanism, which leads to rapid stomatal closure. Both long term and short term of ABA responses enhance plant drought resistance.

In summary, our findings indicate that BnaRGA proteins play important roles in plant adaptation to water-deficit stress. We proposed a working model for BnaA6.RGA-mediated drought tolerance in *B. napus* (Figure 8). Drought leads to an ABA accumulation and a GA reduction, which induces the expression of ABF2 and promotes the RGA accumulation. The BnaA6.RGA-BnaABF2 complex enhances the expression of drought responsive genes (such as *BnaRAB18*), resulting in enhanced ABA signaling, which then increases the plant’s drought resistance. In addition, the accumulated BnaA6.RGA might enhance guard cell sensitivity to ABA by an unknown mechanism, which leads to rapid stomatal closure. Both long term and short term of ABA responses enhance plant drought resistance (Figure 8). Our findings provide novel insights into the crosstalk between GA and ABA signaling pathways, and the editing resources obtained in our study provide desirable germplasm for further breeding of drought tolerance in rapeseed.

## Experimental Procedures

### Plant Materials and Growth Condition

The single mutants, *bnaa6.rga*, *bnac7.rga*, *bnaa9.rga, bnac9.rga*, quadruple mutant *bnarga*, and gain-of-function mutant *bnaa6.rga-D* were generated by the CRISPR/Cas9 technology in *B. napus* (*Westar*) (Yang et al., 2017). The gain-of-function mutants of *BnaC7.RGA*, *ds-3*, was obtained by screening rapeseed EMS library (Zhao et al., 2017). To germinate, the seeds were soaked in water for 7 days and then placed in soil. All plants were cultivated in a growth room under a light intensity of 120 μmol m^–2^ s^–1^ with a 16/8 h light/dark photoperiod (23 ± 1°C and 60–70% RH).

### Plasmid Construction

Each gene was amplified from the *B. napus* cDNA library by PCR using gene-specific primers. For the yeast two hybrid assay, the BnaA10.ABF2 coding region was cloned into the vector *pGBKT7* as the bait (Clontech, United States), while the constructs of *pGADT7-BnaA6/A9/C7/C9.RGA* serving as the prey were obtained from the previous report (Zhao et al., 2017). For the BiFC assay, the full-length coding regions of *BnaRGAs*, and the *N* terminus of *BnaA6.RGA* (1–148 amino acid)/*BnaC7.RGA* (1–132 amino acid), and the *C* terminus of *BnaA6.RGA* (149–572 amino acid)/*BnaC7.RGA* (133–556 amino acid) were cloned into the vector *pFGC-YN173* fused with the *N* terminus of YFP, and *BnaA10.ABF2* was inserted into the vector *pFGC-YC155* fused with the *C* terminus of YFP. For the dual luciferase assay, the full-length coding regions of *BnaA6.RGA*, *BnaC7.RGA*, *BnaA10.ABF2*, and the *N* terminus of *BnaA6.RGA* (1–148 amino acid)/*BnaC7.RGA* (1–132 amino acid) were inserted into the vector *pRI101-GFP* as the effectors, while the 1,496 bp promoter of *BnaC9.RAB18* was cloned into the vector *pGreenII 0800-LUC* as the reporter. All primers are listed in Supplementary Table S1.

### Measurement of Water Loss

To measure the water loss, the leaves were detached from 3-week-old plants and placed in a petri dish on a laboratory bench (23 ± 1°C and 30–40% RH), and the weight of the detached leaves was then measured every 0.5 h for a period of 3 h. The experiment was repeated three times. For each repeat, at least five leaves from different plants of each genotype were used. Water loss was presented as the percentage of the fresh weight (FW) loss.

### Drought Treatment and Relative Water Content

One-week-old plants were transferred from the Hoagland medium to the same weight of water-saturated soil for growing in the greenhouse for 2 weeks, and then deprived of water for 20 days. Each pot contained 130 ± 0.1 g (dry weight, DW) of soil and one plant. The pots were irrigated with water to saturation, allowed to drain, and then weighted to obtain the initial weight, after which they were subjected to drought for different time periods. The relative soil water content (SWC) was calculated as: SWC = (final FW − DW)/(initial weight − DW) × 100. Subsequently, the survival rates of plants were determined after three days of re-watering (rehydration).

The RWC of fully expanded leaves from 3-week-old plants grown in pots was measured after 3 weeks of drought treatment. Frist, leaves were removed and immediately weighted to obtain the FW. The leaves were then placed into petri dishes filled with ddH_2_O. After 24 h, the leaves were blotted to remove external water, and then weighted to obtain the leaf turgid weight (TW). Finally, leaves were dried to a constant weight at 60°C and then weighted to obtain the leaf DW. The RWC of leaves was calculated as: RWC = (FW − DW)/(TW − DW) × 100.

### Measurement of Stomatal Aperture

Stomatal assays were performed as previously described (Desikan et al., 2002). In brief, five fully expanded leaves were harvested from different 3-week-old plants for each genotype and incubated in the MES-KCl buffer (50 mM KCl, 10 mM CaCl_2_, 10 mM MES-KOH, pH 6.15) under light at 22°C for 3 h. Once the stomata were fully open, leaves were incubated in the MES-KCl buffer with 1 μM of ABA or DMSO (control). After 1–2 h of treatment, the epidermal strips were immediately peeled from the abaxial side of leaves. After that, 30–40 stomata from one leaf were measured, adding up to 150–200 stomata for each genotype. The pictures were taken by microscope equipped with a digital camera (AxioCam ICc5, Zeiss). Stomatal aperture was analyzed using the software Image J. Three independent repeats were performed.

### Abscisic Acid Assay

Abscisic acid contents were measured as previously described (Ding et al., 2011). In brief, leaves were harvested from plants that were irrigated or non-irrigated for 10 days. The leaves (about 200 mg) were ground in liquid N_2_ and homogenized in 90% (v/v) methanol containing 200 mg L^–1^ of diethydithiocarbamic acid sodium salt. The extracts were then incubated overnight in darkness at 4°C, followed by a 8,000 × *g* centrifugation at 4°C. The methanolic supernatant was vacuumed centrifuge at 4°C to evaporate the supernatant, and the residue was dissolved by methanolic Tris buffer (10% methanol, 50 mM Tris, pH 8.0, 1 mM MgCl_2_, and 150 mM NaCl). An ELISA kit was used for the determination of ABA following the manufacturer’s instructions (Agdia^1^). Three independent repeats were performed.

### Measurement of Ion Leakage and Malondialdehyde

The membrane ion leakage was measured as described previously with slight modifications (Lee et al., 2011). In brief, five fully expanded leaves, that were irrigated or non-irrigated for 10 or 20 days, were harvested from *bnaa6.rga-D*, *ds-3*, *bnarga*, and their relative wild-type plants. Then, a hole puncher was used to obtain 60 leaf disks from each leaf. The leaf disks were transferred to glass test tubes filled with 10 mL of ddH_2_O, and vacuumed until the leaf disks were submerged by water. After that, the tubes were shaken on an oscillator for 1 h. Electrical conductivity was measured in accordance with the manufacturer’s instructions (BANTE, China). The initial conductivity was measured as R1. Total conductivity was determined after boiling for 10 min as R2. The relative ion leakage was calculated by using the following formula: R1/R2 × 100%.

Malondialdehyde content, expressed as units/mg protein, was measured using analytical kits (Cat#A003-2, Nanjing Jiancheng Bioengineering Institute, Nanjing, China). In all experiments, three independent repeats were performed.

### RNA Extraction and Reverse-Transcription PCR

For transient ABA or drought treatment, the 3-week-old seedlings were transferred into Hoagland medium supplemented with 100 μM ABA or placed in a petri dish on a laboratory bench, respectively. Then the samples were collected at indicated time point, and were frozen immediately by liquid nitrogen. Total RNA was extracted using a Plant Total RNA Isolation Kit (Sangon Biotech, Shanghai, China, No. SK8631) following the manufacturer’s instructions. Approximately 1 μg of total RNA was used for cDNA synthesis using a PrimeScript^TM^ RT reagent kit (TaKaRa, Japan, Cat#RR047A). For qPCR, a total volume of 10 μL reaction mixture was used containing 5 μL of 2 × SYBR Green Master Mix (BioRad, United States), 0.5 μL of 5 × diluted cDNA, 0.25 μL of each primer, and 4 μL of ddH_2_O. Amplification was performed using a CFX Connect^TM^ system (Bio-rad, United States). The amplification program consisted of one cycle of 95°C for 5 min, followed by 50 cycles of 95°C for 15 s, 60°C for 20 s, and 72°C for 20 s. The fluorescent product was detected at the third step of each cycle. The expression level of each gene was calculated using the 2^–ΔΔCT^ method (Livak and Schmittgen, 2001). All analyses were repeated three times using biological replicates. The gene *BnaGAPDH* (BnaC05g12400D) was used as the internal control. All primers are listed in Supplementary Table S2.

### Yeast Two Hybrid

The yeast *Gal4* system was employed for two-hybrid analysis of BnaRGAs and BnaA10.ABF2 protein interactions following the yeast transformation handbook (Yeast Transformation System 2; Clontech, United States). In brief, a single colony of yeast AH109 was incubated at 30°C overnight. The cells were harvested by centrifugation and then resuspended in 25 mL ddH_2_O. Then the cell pellets were dissolved into 1.5-mL sterile 1 × Tris/LiAc solution to make the competent cells. For the yeast two-hybrid assay, the bait (0.5 μg) and/or prey (0.5 μg) plasmids with 0.1 mg of carrier DNA were co-transformed into the yeast-competent cells using polyethylene glycol/LiAc solution. After incubation at 30°C for 30 min, 70 μL DMSO (dimethyl sulfoxide) was added and incubation continued at 42°C for 15 min. The cells were centrifuged and washed using ddH_2_O. The presence of transgenes in yeast cells was confirmed by growing these cells on plates containing solid synthetic defined (SD) medium lacking Leu and Trp (SD/−2). To assess protein–protein interactions, transformed yeast cells were re-suspended in ddH_2_O to an optical density at OD_600_ of 1.0. Samples (5 μL) of suspended yeast cells were spread on plates containing SD medium lacking Ade, His, Leu, and Trp (SD/−4). To detect protein–protein interactions, plates were examined after 3 days of incubation at 30°C. For each experiment, a total of 10 clones were selected and tested.

### Bimolecular Fluorescence Complementation

The BiFC assay was performed as previously described (Koo et al., 2017). In brief, the *Agrobacterium tumefaciens* (strain *GV3101*) cells containing the desired constructs were injected into the 4- to 5-week-old *Nicotiana benthamiana* leaves with the buffer (10 mM MES, pH = 5.7, 10 mM MgCl_2_, and 150 μM acetosyringone). The final concentrations of the bacteria were adjusted to an OD_600_ = 0.2 for each construct. The transiently transformed leaves were analyzed at 48 h after injection. The YFP fluorescence (excitation/emission wavelength: 514 nm/527 nm) was observed under a fluorescence microscope (SPX8, Leica).

### Dual-Luciferase Assay

Dual-luciferase assays were performed by using the Dual-Luciferase_®_ Reporter Assay System (Promega, Madison, WI, United States). All reagents were prepared as described by the manufacturer. Briefly, the agrobacteria harboring the reporter and effector vectors were injected into the tobacco leaves. After 3 days, leaf disks in a diameter of 2 cm were harvested and ground, and dissolved into 100 μL of Passive Lysis Buffer. After 30 s, a 50 μL aliquot was used for luminescence measurements with the SPARK^®^ MULTIMODE MICROPLATE (TECAN, Swiss). The following steps were used for luminescence measurements: 50 μL of the firefly luciferase reagent (LARII) was added to the test sample, with a 10 s equilibration time and measurement of luminescence with a 10 s integration time, followed by addition of 50 μl of the REN reagent and firefly quenching (Stop and Glow^TM^ buffer), 10 s equilibration time, and measurement of luminescence with a 10 s integration time. The data are represented as the ratio of firefly to Renilla luciferase activity (Fluc/Rluc). Each data point consisted of at least three biological replicates, and 10 repeats were performed for each assay.

### Phylogenetic Analysis

The protein sequences were obtained from the website^2^ (Jin et al., 2017). The sequence alignment was performed using Clustal Omega.^3^ An unrooted phylogenetic tree was constructed using MEGA7^4^ (Kumar et al., 2016) with the neighbor-joining statistical method and bootstrap analysis (1,000 replicates).

### Statistical Analyses

Statistical analyses were performed to determine significant differences between genotypes, using Duncan’s multiple range tests in the ANOVA program of SPSS (IBM SPSS 22). at *P* values <0.05 or <0.01.

## Data Availability Statement

Sequence data from this article can be found in the *Brassica napus* Genome database (https://plants.ensembl.org/Brassica_napus/Info/Index) under the following accession numbers: BnaA10.ABF2 (BnaA10g28780D), BnaC9.RAB18 (BnaC09g08130D), BnaRD29A (BnaC03g15510D), BnaRD29B (BnaA03g12660D), BnaA9.RGA (BnaA09g18700D), BnaC7.RGA (BnaC07g20900D), BnaA6.RGA (BnaA06g34810D), BnaC9.RGA (BnaC09g52270D), BnaC6.ABF2 (BnaC06g00420D), BnaABA1 (BnaA09g07610D), BnaABA2 (BnaC06g41140D), BnaGID1a (BnaA05g32040D), BnaGID1c (BnaA09g20650D), BnaSLY1 (BnaA01g13690D), BnaSLY2 (BnaC02g38380D), BnaGAPDH (BnaC05g12400D).

## Author Contributions

CD and JW designed the research. JW, ZD, GY, and ZW performed the experiments. KL, LG, JT, JS, BY, TF, and CM provided lab support. CD, JW, and GY analyzed the data. CD, CK, JW, and ZD wrote the manuscript. All authors read and approved the manuscript.

## Conflict of Interest

The authors declare that the research was conducted in the absence of any commercial or financial relationships that could be construed as a potential conflict of interest.

## Footnotes

1 http://www.agdia.com

2 http://planttfdb.cbi.pku.edu.cn/index.php

3 http://www.ebi.ac.uk/Tools/msa/clustalo

4 http://www.megasoftware.net/

## References

[B1] Bai M. Y. Shang J. X. Oh E. Fan M. Bai Y. Zentella R. (2012). Brassinosteroid, gibberellin and phytochrome impinge on a common transcription module in *Arabidopsis*. *Nat. Cell. Biol.* 14 810–817. 10.1038/ncb2546 22820377 PMC3606816

[B2] Chalhoub B. Denoeud F. Liu S. Parkin I. A. Tang H. Wang X. (2014). Plant genetics. Early allopolyploid evolution in the post-Neolithic *Brassica napus* oilseed genome. *Science* 345 950–953. 10.1126/science.1253435 25146293

[B3] Chaves M. Maroco J. Pereira J. (2003). Understanding plant responses to drought - From genes to the whole plant. *Funct. Plant Biol.* 30 239–264. 10.1071/FP02076 32689007

[B4] Chen K. Li G. J. Bressan R. A. Song C. P. Zhu J. K. Zhao Y. (2020). Abscisic acid dynamics, signaling, and functions in plants. *J. Integr. Plant Biol* 62 25–54. 10.1111/jipb.12899 31850654

[B5] Colebrook E. H. Thomas S. G. Phillips A. L. Hedden P. (2014). The role of gibberellin signalling in plant responses to abiotic stress. *J. Exp. Biol.* 217 67–75. 10.1242/jeb.089938 24353205

[B6] Dalal M. Tayal D. Chinnusamy V. Bansal K. C. (2009). Abiotic stress and ABA-inducible Group 4 LEA from *Brassica napus* plays a key role in salt and drought tolerance. *J. Biotechnol.* 139 137–145. 10.1016/j.jbiotec.2008.09.014 19014980

[B7] de Lucas M. Daviere J. M. Rodriguez-Falcon M. Pontin M. Iglesias-Pedraz J. M. Lorrain S. (2008). A molecular framework for light and gibberellin control of cell elongation. *Nature* 451 480–484. 10.1038/nature06520 18216857

[B8] Desikan R. Griffiths R. Hancock J. Neill S. (2002). A new role for an old enzyme: nitrate reductase-mediated nitric oxide generation is required for abscisic acid-induced stomatal closure in *Arabidopsis thaliana*. *Proc. Natl. Acad. Sci. U.S.A.* 99 16314–16318. 10.1073/pnas.252461999 12446847 PMC138608

[B9] Ding Y. Avramova Z. Fromm M. (2011). The *Arabidopsis trithorax*-like factor ATX1 functions in dehydration stress responses via ABA-dependent and ABA-independent pathways. *Plant J.* 66 735–744. 10.1111/j.1365-313X.2011.04534.x 21309869

[B10] Fleet C. M. Sun T. P. (2005). A DELLAcate balance: the role of gibberellin in plant morphogenesis. *Curr. Opin. Plant Biol.* 8 77–85. 10.1016/j.pbi.2004.11.015 15653404

[B11] Fujita Y. Fujita M. Satoh R. Maruyama K. Parvez M. M. Seki M. (2005). AREB1 is a transcription activator of novel ABRE-dependent ABA signaling that enhances drought stress tolerance in *Arabidopsis*. *Plant Cell* 17 3470–3488. 10.1105/tpc.105.035659 16284313 PMC1315382

[B12] Good A. G. Zaplachinski S. T. (1994). The effects of drought stress on free amino acid accumulation and protein synthesis in *Brassica napus*. *Physiol. Plant.* 90 9–14.

[B13] Hirano K. Kouketu E. Katoh H. Aya K. Ueguchi-Tanaka M. Matsuoka M. (2012). The suppressive function of the rice DELLA protein SLR1 is dependent on its transcriptional activation activity. *Plant J.* 71 443–453. 10.1111/j.1365-313X.2012.05000.x 22429711

[B14] Hirsch S. Oldroyd G. E. (2009). GRAS-domain transcription factors that regulate plant development. *Plant Signal. Behav.* 4 698–700. 10.4161/psb.4.8.9176 19820314 PMC2801379

[B15] Hossain M. A. Cho J. I. Han M. Ahn C. H. Jeon J. S. An G. (2010). The ABRE-binding bZIP transcription factor OsABF2 is a positive regulator of abiotic stress and ABA signaling in rice. *J. Plant Physiol.* 167 1512–1520. 10.1016/j.jplph.2010.05.008 20576316

[B16] Hou X. Lee L. Y. Xia K. Yan Y. Yu H. (2010). DELLAs modulate jasmonate signaling via competitive binding to JAZs. *Dev. Cell.* 19 884–894. 10.1016/j.devcel.2010.10.024 21145503

[B17] Illouz-Eliaz N. Ramon U. Shohat H. Blum S. Livne S. Mendelson D. (2019). Multiple gibberellin receptors contribute to phenotypic stability under changing environments. *Plant Cell* 31 1506–1519. 10.1105/tpc.19.00235 31076539 PMC6635849

[B18] Jin J. Tian F. Yang D. C. Meng Y. Q. Kong L. Luo J. (2017). PlantTFDB 4.0: toward a central hub for transcription factors and regulatory interactions in plants. *Nucleic Acids Res.* 45 D1040–D1045. 10.1093/nar/gkw982 27924042 PMC5210657

[B19] Kilian J. Whitehead D. Horak J. Wanke D. Weinl S. Batistic O. (2007). The AtGenExpress global stress expression data set: protocols, evaluation and model data analysis of UV-B light, drought and cold stress responses. *Plant J.* 50 347–363. 10.1111/j.1365-313X.2007.03052.x 17376166

[B20] Koo J. C. Lee I. C. Dai C. Lee Y. Cho H. K. Kim Y. (2017). The protein trio RPK1-CaM4-RbohF mediates transient superoxide production to trigger age-dependent cell death in *Arabidopsis*. *Cell Rep.* 21 3373–3380. 10.1016/j.celrep.2017.11.077 29262318

[B21] Kumar S. Stecher G. Tamura K. (2016). MEGA7: molecular evolutionary genetics analysis Version 7.0 for bigger datasets. *Mol. Biol. Evol.* 33 1870–1874. 10.1093/molbev/msw054 27004904 PMC8210823

[B22] Lee I. C. Hong S. W. Whang S. S. Lim P. O. Nam H. G. Koo J. C. (2011). Age-dependent action of an ABA-inducible receptor kinase, RPK1, as a positive regulator of senescence in *Arabidopsis* leaves. *Plant Cell Physiol.* 52 651–662. 10.1093/pcp/pcr026 21382977

[B23] Li F. Wu X. Tsang E. Cutler A. J. (2005). Transcriptional profiling of imbibed *Brassica napus* seed. *Genomics* 86 718–730. 10.1016/j.ygeno.2005.07.006 16125897

[B24] Li X. Y. Liu X. Yao Y. Li Y. H. Liu S. He C. Y. (2013). Overexpression of Arachis hypogaea AREB1 gene enhances drought tolerance by modulating ROS scavenging and maintaining endogenous ABA content. *Int. J. Mol. Sci.* 14 12827–12842. 10.3390/ijms140612827 23783278 PMC3709814

[B25] Lim S. Park J. Lee N. Jeong J. Toh S. Watanabe A. (2013). ABA-insensitive3, ABA-insensitive5, and DELLAs Interact to activate the expression of SOMNUS and other high-temperature-inducible genes in imbibed seeds in *Arabidopsis*. *Plant Cell* 25 4863–4878. 10.1105/tpc.113.118604 24326588 PMC3903992

[B26] Livak K. J. Schmittgen T. D. (2001). Analysis of relative gene expression data using real-time quantitative PCR and the 2^−ΔΔC_T_^ Method. *Methods* 25, 402–408. 10.1006/meth.2001.1262 11846609

[B27] Ma Y. Szostkiewicz I. Korte A. Moes D. Yang Y. Christmann A. (2009). Regulators of PP2C phosphatase activity function as abscisic acid sensors. *Science* 324 1064–1068. 10.1126/science.1172408 19407143

[B28] Magome H. Yamaguchi S. Hanada A. Kamiya Y. Oda K. (2008). The DDF1 transcriptional activator upregulates expression of a gibberellin-deactivating gene, GA2ox7, under high-salinity stress in *Arabidopsis*. *Plant J.* 56 613–626. 10.1111/j.1365-313X.2008.03627.x 18643985

[B29] McAdam S. A. M. Brodribb T. J. (2016). Linking turgor with ABA biosynthesis: implications for stomatal responses to vapor pressure deficit across land plants. *Plant Physiol.* 171 2008–2016. 10.1104/pp.16.00380 27208264 PMC4936570

[B30] Müller T. Lentzsch P. Müller M. E. H. (2012). Carbohydrate dynamics in leaves of rapeseed (*Brassica napus*) under drought. *J. Agron. Crop Sci.* 198 207–217.

[B31] Mustilli A. C. Merlot S. Vavasseur A. Fenzi F. Giraudat J. (2002). *Arabidopsis* OST1 protein kinase mediates the regulation of stomatal aperture by abscisic acid and acts upstream of reactive oxygen species production. *Plant Cell* 14 3089–3099. 10.1105/tpc.007906 12468729 PMC151204

[B32] Nakamura H. Xue Y. L. Miyakawa T. Hou F. Qin H. M. Fukui K. (2013). Molecular mechanism of strigolactone perception by DWARF14. *Nat. Commun.* 4:2613. 10.1038/ncomms3613 24131983

[B33] Nelissen H. Sun X.-H. Rymen B. Jikumaru Y. Kojima M. Takebayashi Y. (2018). The reduction in maize leaf growth under mild drought affects the transition between cell division and cell expansion and cannot be restored by elevated gibberellic acid levels. *Plant Biotechnol. J.* 16 615–627. 10.1111/pbi.12801 28730636 PMC5787831

[B34] Nir I. Moshelion M. Weiss D. (2014). The *Arabidopsis gibberellin* methyl transferase 1 suppresses gibberellin activity, reduces whole-plant transpiration and promotes drought tolerance in transgenic tomato. *Plant Cell Environ.* 37 113–123. 10.1111/pce.12135 23668385

[B35] Nir I. Shohat H. Panizel I. Olszewski N. Aharoni A. Weiss D. (2017). The tomato DELLA protein PROCERA acts in guard cells to promote stomatal closure. *Plant Cell* 29 3186–3197. 10.1105/tpc.17.00542 29150547 PMC5757276

[B36] Park S. Y. Fung P. Nishimura N. Jensen D. R. Fujii H. Zhao Y. (2009). Abscisic acid inhibits type 2C protein phosphatases via the PYR/PYL family of START proteins. *Science* 324 1068–1071. 10.1126/science.1173041 19407142 PMC2827199

[B37] Piskurewicz U. Jikumaru Y. Kinoshita N. Nambara E. Kamiya Y. Lopez-Molina L. (2008). The gibberellic acid signaling repressor RGL2 inhibits *Arabidopsis* seed germination by stimulating abscisic acid synthesis and ABI5 activity. *Plant Cell* 20 2729–2745. 10.1105/tpc.108.061515 18941053 PMC2590721

[B38] Qaderi M. M. Kurepin L. V. Reid D. M. (2006). Growth and physiological responses of canola (*Brassica napus*) to three components of global climate change: temperature, carbon dioxide and drought. *Physiol. Plant.* 128 710–721.

[B39] Rodriguez-Leal D. Lemmon Z. H. Man J. Bartlett M. E. Lippman Z. B. (2017). Engineering quantitative trait variation for crop improvement by genome editing. *Cell* 171 470.e8–480.e8. 10.1016/j.cell.2017.08.030 28919077

[B40] Soon F. F. Ng L. M. Zhou X. E. West G. M. Kovach A. Tan M. H. (2012). Molecular mimicry regulates ABA signaling by SnRK2 kinases and PP2C phosphatases. *Science* 335 85–88. 10.1126/science.1215106 22116026 PMC3584687

[B41] Ueguchi-Tanaka M. Nakajima M. Katoh E. Ohmiya H. Asano K. Saji S. (2007). Molecular interactions of a soluble gibberellin receptor, GID1, with a rice DELLA protein, SLR1, and gibberellin. *Plant Cell* 19 2140–2155. 10.1105/tpc.106.043729 17644730 PMC1955699

[B42] Van De Velde K. Ruelens P. Geuten K. Rohde A. Van Der Straeten D. (2017). Exploiting DELLA signaling in cereals. *Trends Plant Sci.* 22 880–893. 10.1016/j.tplants.2017.07.010 28843766

[B43] Vishal B. Kumar P. P. (2018). Regulation of seed germination and abiotic stresses by gibberellins and abscisic acid. *Front. Plant Sci.* 9:838. 10.3389/fpls.2018.00838 29973944 PMC6019495

[B44] Wang Z. Liu L. Cheng C. Ren Z. Xu S. Li X. (2020). GAI functions in the plant response to dehydration stress in *Arabidopsis thaliana*. *Int. J. Mol. Sci.* 21:819. 10.3390/ijms21030819 32012796 PMC7037545

[B45] Xiong L. Schumaker K. S. Zhu J. K. (2002). Cell signaling during cold, drought, and salt stress. *Plant Cell* 14(Suppl.), S165–S183. 10.1105/tpc.000596 12045276 PMC151254

[B46] Yang H. Wu J. J. Tang T. Liu K. D. Dai C. (2017). CRISPR/Cas9-mediated genome editing efficiently creates specific mutations at multiple loci using one sgRNA in *Brassica napus*. *Sci. Rep.* 7:7489. 10.1038/s41598-018-23161-4 28790350 PMC5548805

[B47] Yoshida H. Hirano K. Sato T. Mitsuda N. Nomoto M. Maeo K. (2014). DELLA protein functions as a transcriptional activator through the DNA binding of the indeterminate domain family proteins. *Proc. Natl. Acad. Sci. U.S.A.* 111 7861–7866. 10.1073/pnas.1321669111 24821766 PMC4040565

[B48] Zentella R. Zhang Z. L. Park M. Thomas S. G. Endo A. Murase K. (2007). Global analysis of della direct targets in early gibberellin signaling in *Arabidopsis*. *Plant Cell* 19 3037–3057. 10.1105/tpc.107.054999 17933900 PMC2174696

[B49] Zhang D. Jing Y. Jiang Z. Lin R. (2014). The chromatin-remodeling factor PICKLE integrates brassinosteroid and gibberellin signaling during skotomorphogenic growth in *Arabidopsis*. *Plant Cell* 26 2472–2485. 10.1105/tpc.113.121848 24920333 PMC4114946

[B50] Zhao B. Li H. Li J. Wang B. Dai C. Wang J. (2017). *Brassica napus* DS-3, encoding a DELLA protein, negatively regulates stem elongation through gibberellin signaling pathway. *Theor. Appl. Genet.* 130 727–741. 10.1007/s00122-016-2846-4 28093630

[B51] Zhao B. Y. Hu Y. F. Li J. J. Yao X. Liu K. D. (2016). BnaABF2, a bZIP transcription factor from rapeseed (*Brassica napus* L.), enhances drought and salt tolerance in transgenic *Arabidopsis*. *Bot. Stud.* 57:12. 10.1186/s40529-016-0127-9 28597422 PMC5432893

[B52] Zhu J. K. (2002). Salt and drought stress signal transduction in plants. *Annu. Rev. Plant Biol.* 53 247–273. 10.1146/annurev.arplant.53.091401.143329 12221975 PMC3128348

[B53] Zhu J. K. (2016). Abiotic stress signaling and responses in plants. *Cell* 167 313–324. 10.1016/j.cell.2016.08.029 27716505 PMC5104190

[B54] Zhu M. Monroe J. G. Suhail Y. Villiers F. Mullen J. Pater D. (2016). Molecular and systems approaches towards drought-tolerant canola crops. *New Phytol.* 210 1169–1189. 10.1111/nph.13866 26879345

[B55] Zhu M. Simons B. Zhu N. Oppenheimer D. G. Chen S. (2010). Analysis of abscisic acid responsive proteins in *Brassica napus* guard cells by multiplexed isobaric tagging. *J. Proteomics* 73 790–805. 10.1016/j.jprot.2009.11.002 19913118

