# Supplementary material for: Roles of the Brassica napus DELLA Protein BnaA6.RGA, in Modulating Drought Tolerance by Interacting With the ABA Signaling Component BnaA10.ABF2
Source: Front Plant Sci. 2020 May 14;11:577. doi: 10.3389/fpls.2020.00577 (PMC7240051; doi:10.3389/fpls.2020.00577)
Supplement: Supplementary file 1 [file Data_Sheet_1.docx]

**Roles of *Brassica napus* DELLA Protein BnaA6.RGA in modulation of drought tolerance by interacting one of ABA signaling component BnaA10.ABF2**

Jiajing Wu^1*^, Guanbo Yan^1*^, Zhiqiang Duan^1^, Zhijuan Wang^2^, Chunying Kang^3^, Liang Guo^1^, Kede Liu^1^, Jinxing Tu^1^, Jinxiong Shen^1^, Bin Yi^1^, Tingdong Fu^1^, Xia Li^2^, Chaozhi Ma^1#^ and Cheng Dai^1#^

1. National Key Laboratory of Crop Genetic Improvement, Huazhong Agricultural University, Wuhan 430070, China

2. State Key Laboratory of Agricultural Microbiology, College of Plant Science and Technology, Huazhong Agricultural University, Wuhan 430070, China

3. Key Laboratory of Horticultural Plant Biology (Ministry of Education), College of Horticulture and Forestry Sciences, Huazhong Agricultural University, Wuhan 430070, China

**# For whom correspondence should be addressed.**

Dr. Cheng Dai, Email: [cdai@mail.hzau.edu.cn](mailto:cdai@mail.hzau.edu.cn)

Dr. Chaozhi Ma, Email: yuanbeauty@mail.hzau.edu.cn

**
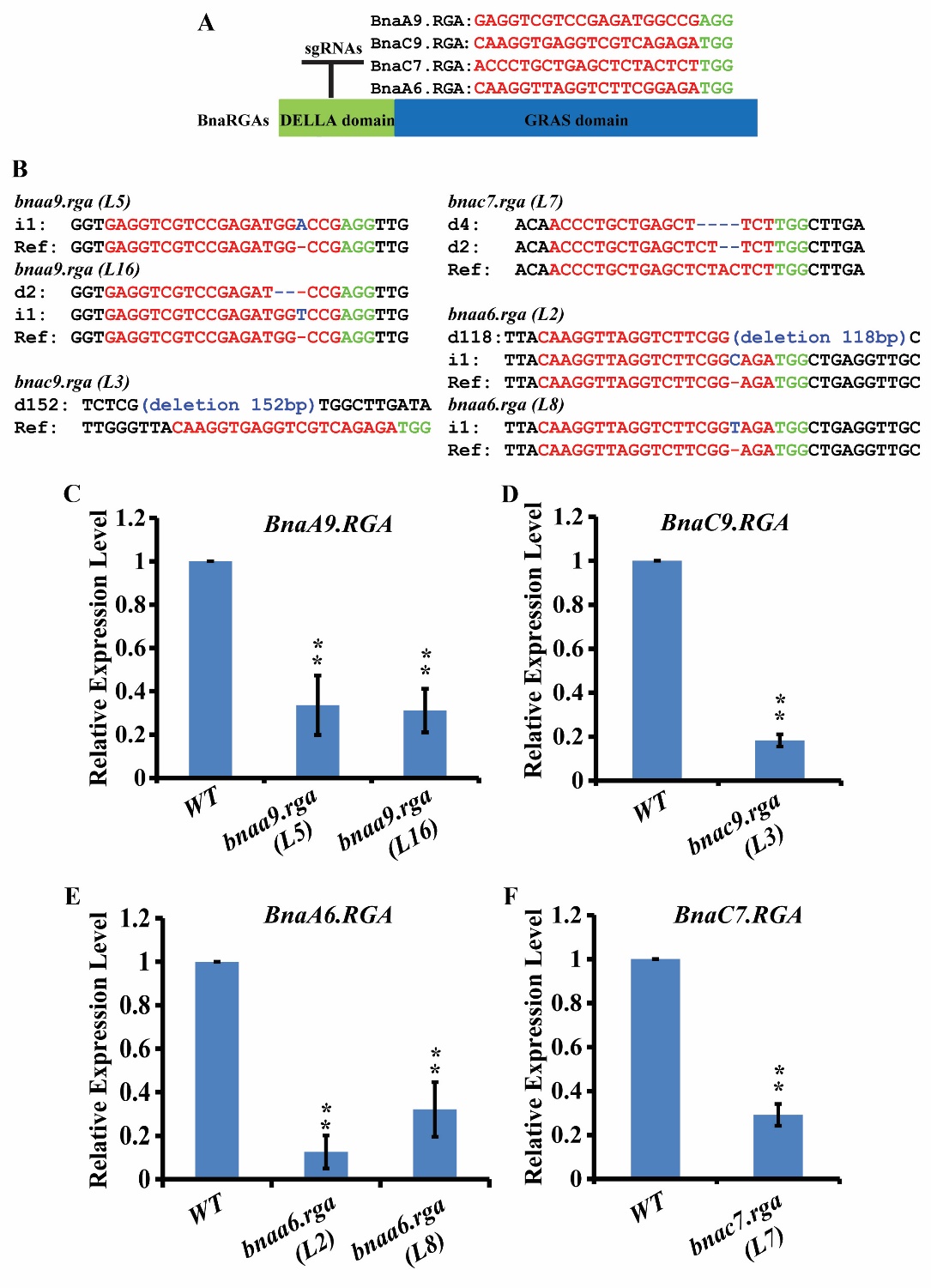
**

**Figure S1. Genotypes and expression of *BnaRGAs* in single mutants created by CRISPR/Cas9.**

(A). Schematic diagram showing the domians in BnaRGAs and the sgRNAs targeted four *BnaRGA* paralogue genes individually. (B). The genotype of the *BnaRGAs* in single mutant lines. The PAM sites (TGG) are indicated with green. In (A) and (B), the PAM sequence is indicated with Green, and the sgRNA is indicated with red. The mutation sites are indicated with blue. d: deletion. d#, # of base pair (bp) deleted from target site. (C) to (F). Expression level of *BnaA9.RGA*, *BnaC9.RGA*, *BnaA6.RGA*, and *BnaC7.RGA* was suppressed in single mutants. *BnaGAPDH* was used as the internal control. Asterisks show that the values are significantly different between the WT and mutants at the same stage. Data are means ± SD obtained from three biological replicates. **, *P* < 0.01, Student’s *t*-test.

**
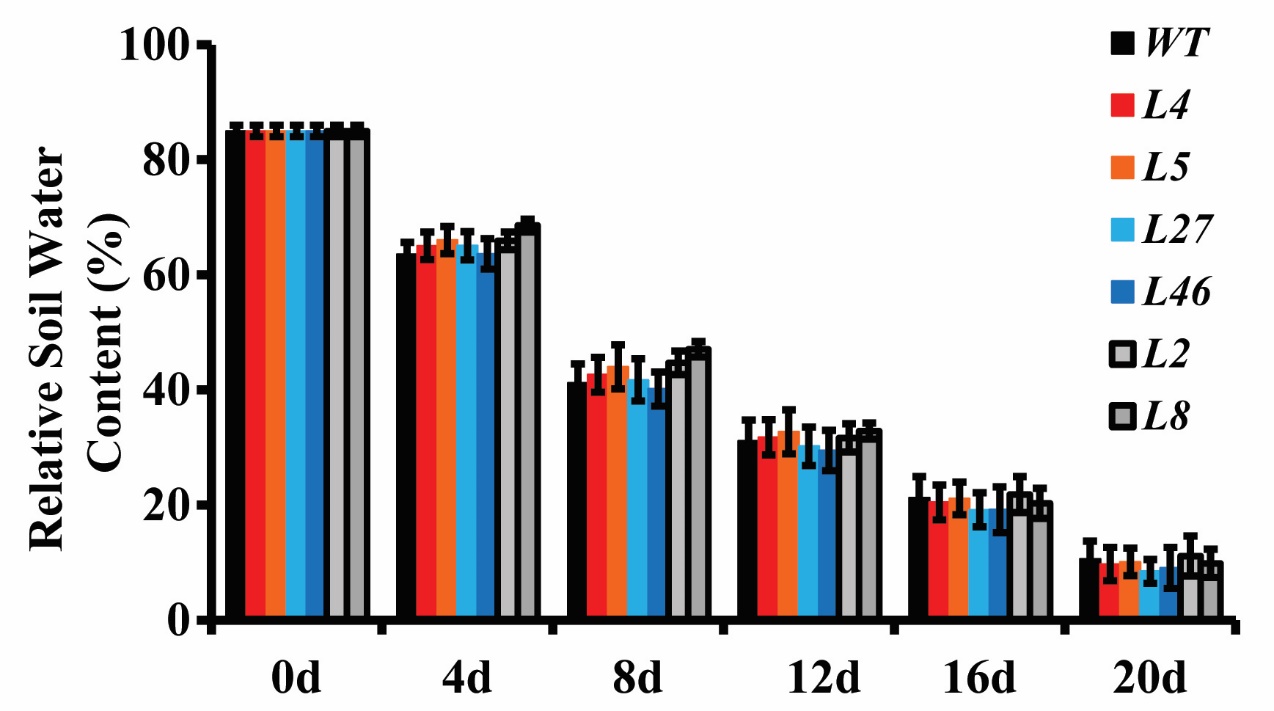
**

**Figure S2. Relative water content of soil after drought treatment.**

Relative water content of soil after drought treatment at the different time point. WT: Westar. L4 and L5: two lines of *bnaa6.rga-D*. L27 and L46: two lines of *bnarga*. L2 and L8: two lines of *bnaa6.rga*.


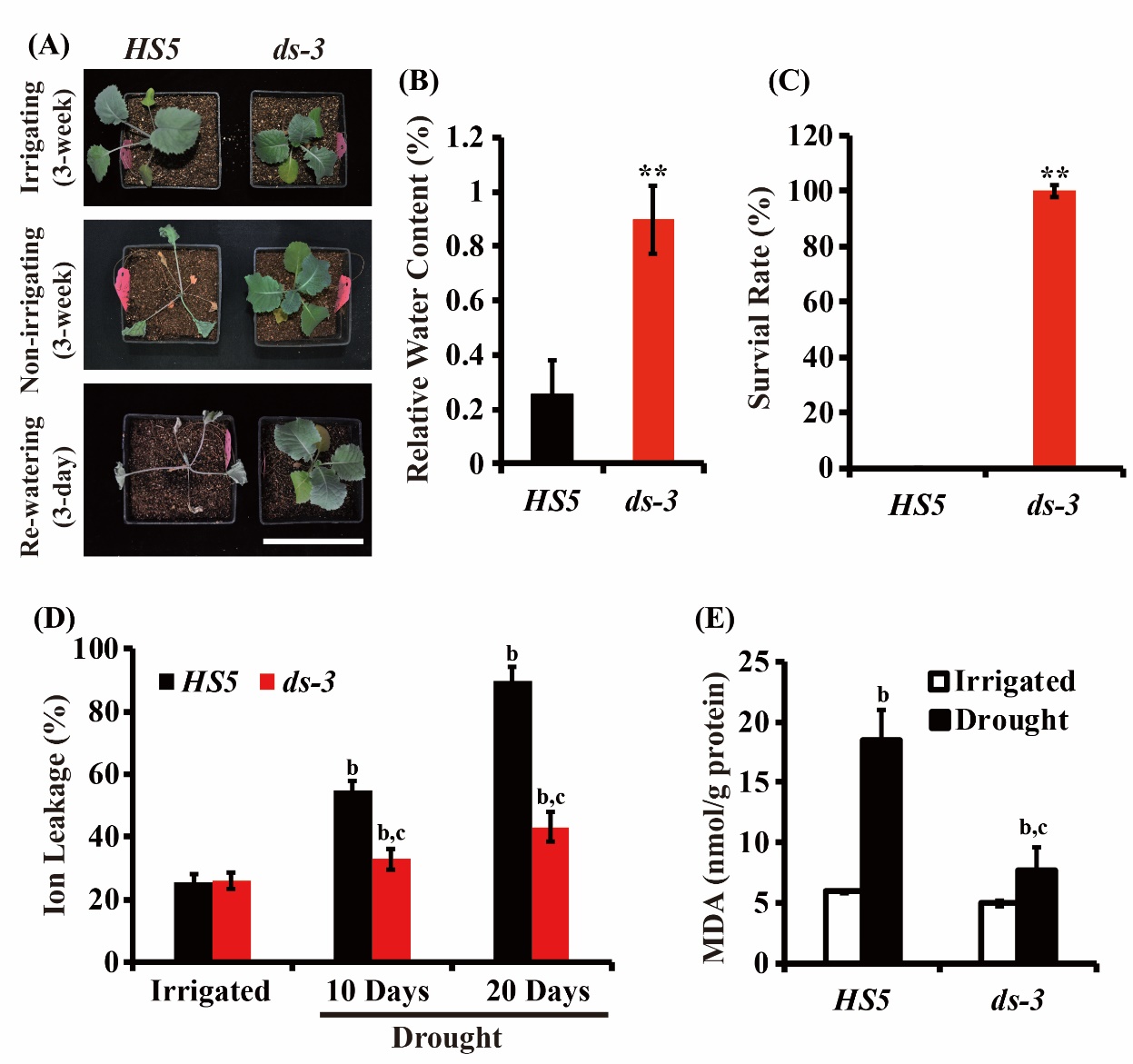


**Figure S3. The survival rate of *ds-3* after drought treatment.**

(A). Images showing the phenotypes of HS5, and *ds-3* in response to progressive drought stress. Images were taken for well-watered plants, at 20 days without irrigation, and at 3 days after rehydration. (B). Bar graph showing the leaf relative water content of HS5, and *ds-3* under water-deficit conditions in (A). (C). Bar graph showing the survival rates of HS5, and *ds-3* under water-deficit conditions followed by re-watering. (D). Bar graph showing the ion leakage of HS5, and *ds-3* in response to progressive drought stress. (E). Bar graph showing the MDA content of WT, *bnaA6.rga-D*, and *bnaa6.aga* before and after drought treatment. In (B), and (C), data are means ± SD (n=10-15) obtained from three biological experiments. Asterisks show that the values are significantly different between the WT and different mutants at the same time point. The data were analyzed by Duncan's multiple range tests in the ANOVA program of SPSS (*, P < 0.05; **, P < 0.01). In (D) and (E), letters indicate statistically significant differences between b: drought treatment vs. control WT, and c: drought treatment of mutants vs. drought treatment of WT at p < 0.05 (Duncan's multiple range tests). In (a)-(e), L4 and L6: two individual *bnaa6.rga-D* lines; L2 and L8: two individual *bnaa6.rga* lines; WT: *Westar*.


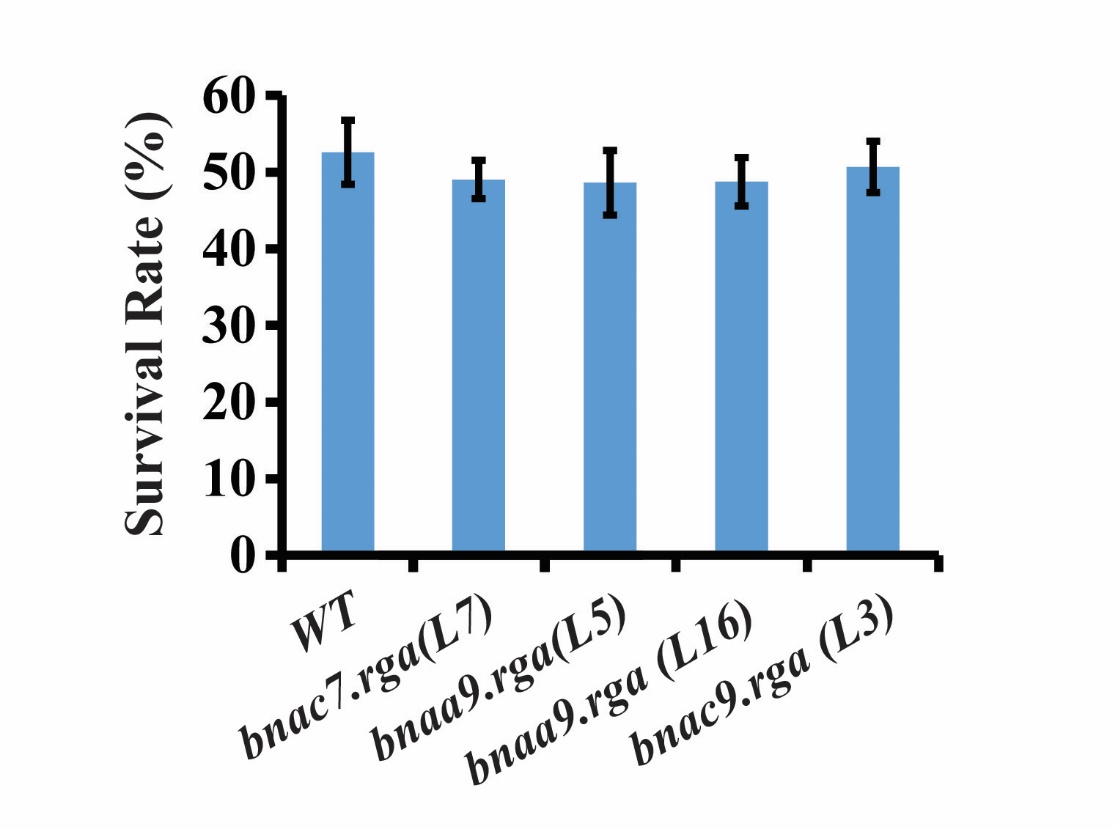


**Figure S4. The survival rate of *BnaRGA* single mutants after drought treatment.**

The bar graph shows the survival rate of WT, *bnaa9.rga*, *bnac9.rga*, *bnac7.rga* after mild drought treatment. Data are means ± SD (n=10) from three individual experiments. No significance was found (Student’s *t*-test, *P* < 0.05).


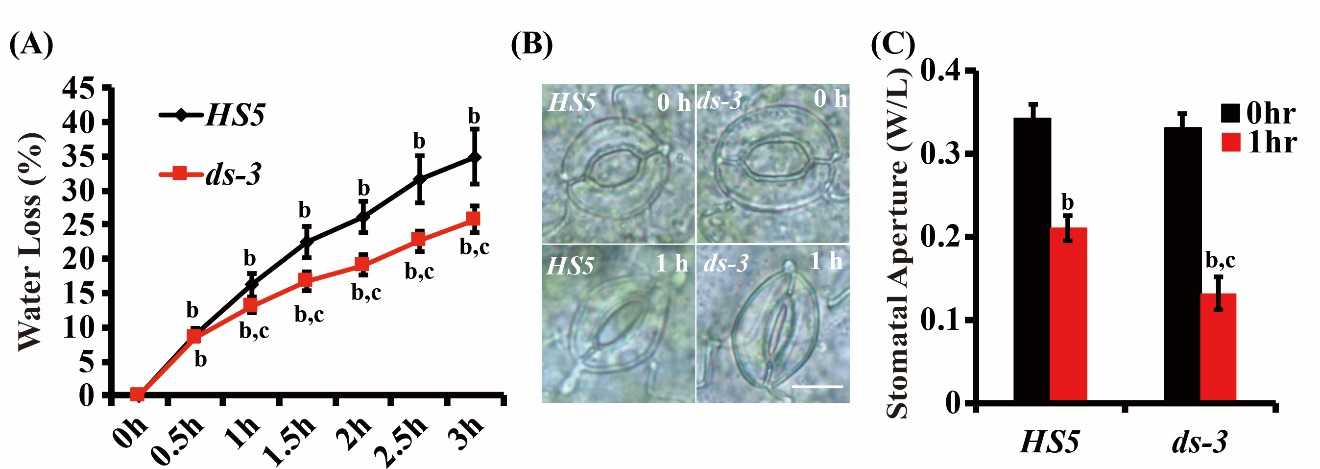


**Figure S5. Water loss of detached leaves and stomatal aperture after ABA treatment of *ds-3*.**

(A). Water loss of detached leaves of HS5, and *ds-3*. Leaves at similar developmental stages were excised and weighed at the indicated time after detachment. The proportion of fresh weight losses was calculated on the basis of the initial weight of the leaves. (B). Images of the representative stomata of *ds-3*, and *HS5* at 0, and 1 h with or without ABA (1 µM) treatment. Scale bars: 10 μm. (C). Line graph showing the stomatal apertures measured from (b). In (A), and (C), data are means ± SD (n=5-6 for (A); n=150-200 for (C)) obtained from three biological experiments. In (A), and (C), letters indicate statistically significant differences between b: drought (or ABA) treatment vs. control WT, and c: drought (or ABA) treatment of mutants vs. drought (or ABA) treatment of WT at p < 0.05 (Duncan's multiple range tests).

**
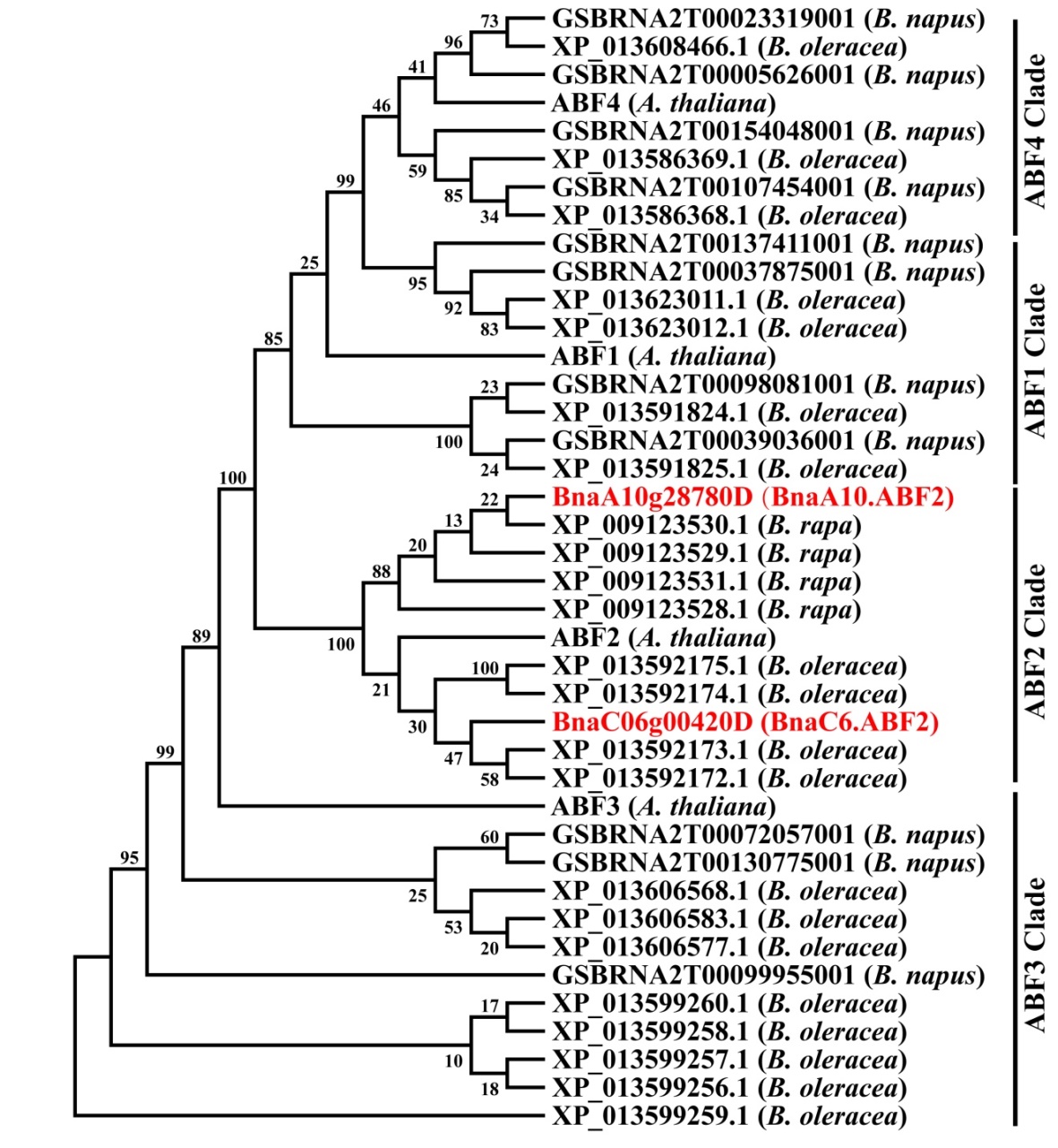
**

**Figure S6. Phylogenetic analysis of the ABF homologs in *Arabidopsis thaliana, Brassica napus*, *Brassica rapa*, and *Brassica oleracea*.**

Four clades were formed in the phylogenetic tree consisting of 40 ABF proteins from *Arabidopsis* (4), *B. napus* (13), *B. rapa* (4), and *B. oleracea* (19). BnaA10.ABF2 and BnaC6.ABF2 are marked with red.


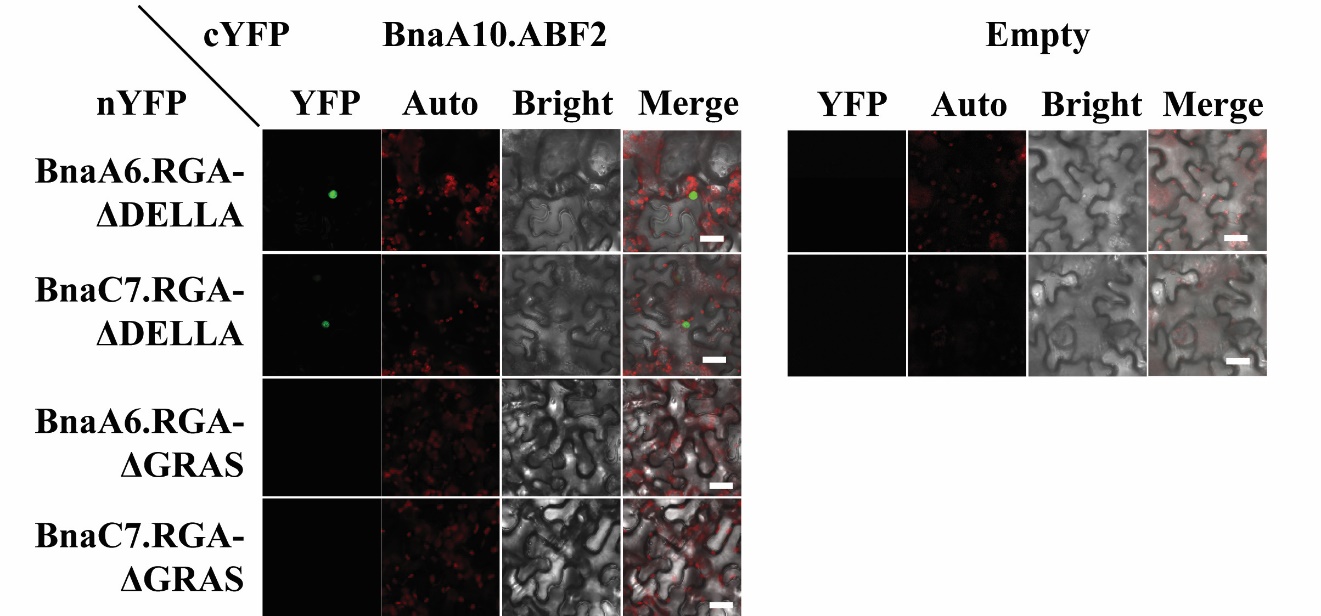


**Figure S7. GRAS domain of BnaRGAs physically interact with BnaA10.ABF2.**

BnaA6.RGA-ΔDELLA and BnaC7.RGA-ΔDELLA interacted with BnaA10.ABF2 in the BiFC assay performed in the *N. benthamiana* leaves. Scale bars: 50 μm. Images were acquired by confocal microscope using the identical settings. YFP: Yellow Fluorescent Protein, Auto: chloroplast auto fluorescence, Bright: Bright Field, Merge: the figure merged by YFP, Auto and Bright.

**
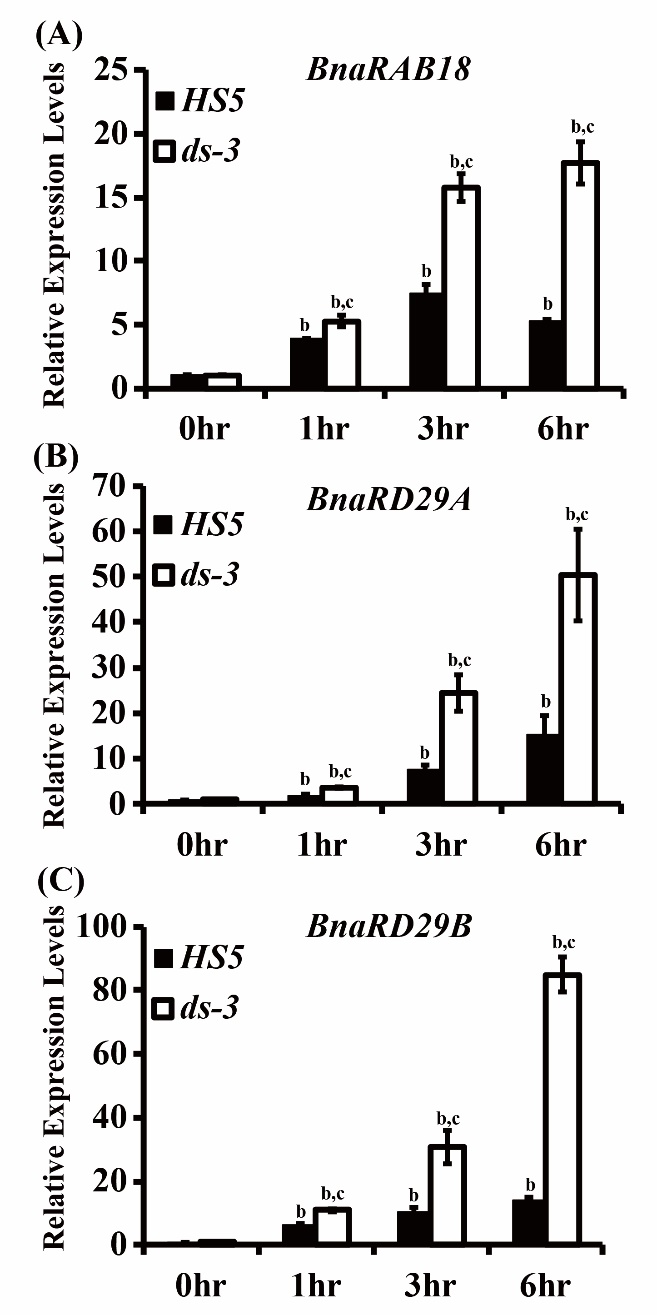
**

**Figure S8. Expression of *BnaRAB18*, *BnaRD29A* and *BnaRD29B* in *ds-3* and *HS5* in response to drought and ABA treatment.**

(A) to (C). Expression levels of *BnaRAB18* (A), *BnaRD29A* (B), and *BnaRD29B* (C) in *ds-3*, and HS5 after drought treatment examined by qRT-PCR. The expressions level of each gene at 0 h was set as 1. *BnaGAPDH* was used as the internal control. Data are means ± SD obtained from three biological replicates. In (A)-(C), letters indicate statistically significant differences between b: drought treatment vs. control WT, and c: drought treatment of mutants vs. drought treatment of WT at p < 0.05 (Duncan's multiple range tests).

**
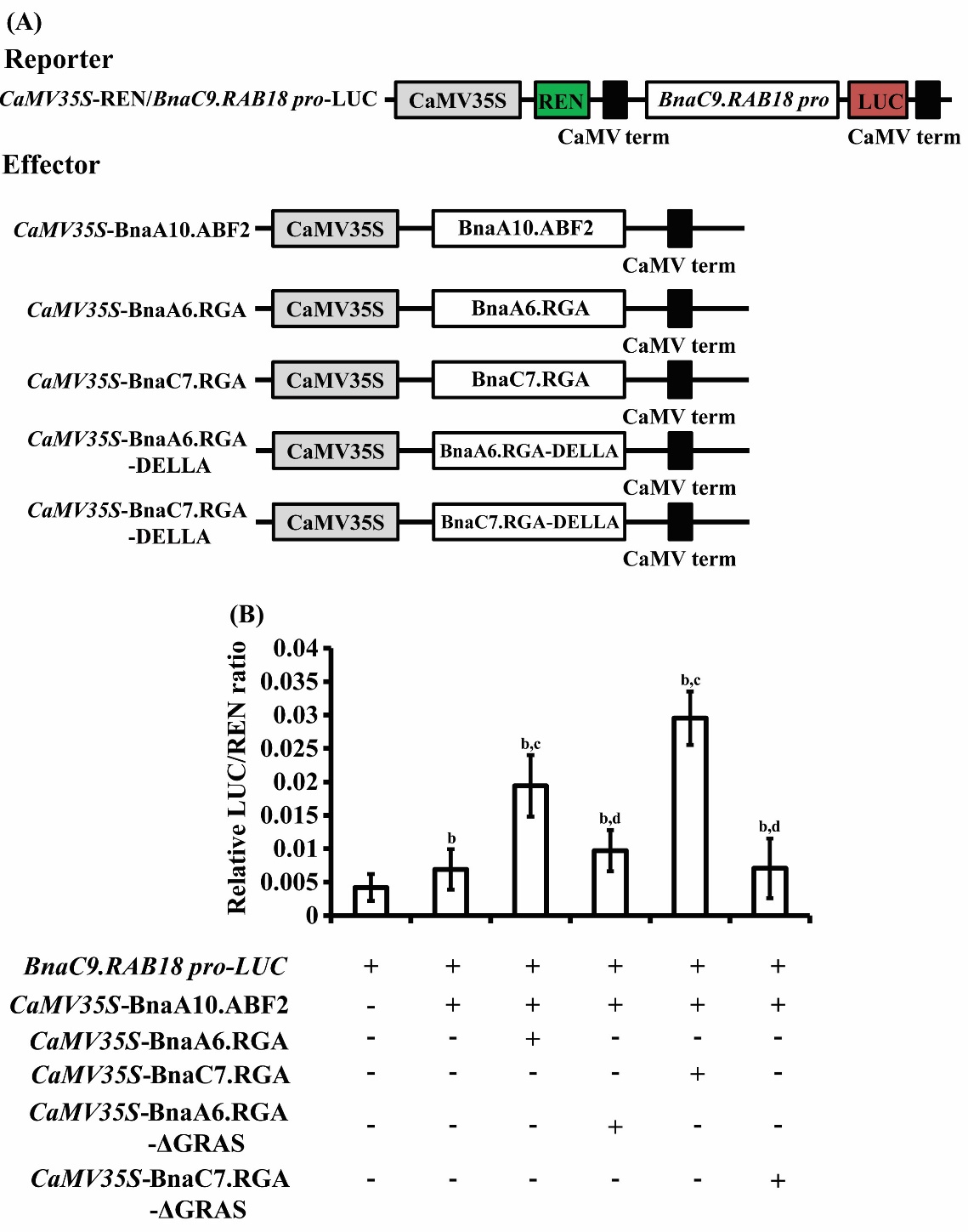
**

**Figure S9. Transcriptional regulation of *BnaC9.RAB18* by BnaA10.ABF2 was not enhanced by truncated BnaA6.RGA and BnaC7.RGA, respectively.**

(A). Schematic representation of the constructs used for the dual-luciferase assay. The reporter construct contains the firefly luciferase driven by *BnaC9.RAB18* promoter, and the Renilla luciferase (REN) driven by the *CaMV 35S promoter*. The effector constructs contain BnaA6.RGA, BnaC7.RGA, BnaA6.RGA-ΔGRAS, BnaC7.RGA-ΔGRAS, and BnaA10.ABF2 driven by the *CaMV 35S* promoter, respectively. (B). Bar graph showing the LUC/REN ratios in the dual-luciferase assay. Data are means ± SD obtained from three biological replicates. In (B), letters indicate statistically significant differences between (b) co-infiltrated effectors with reporters (*BnaC9.RAB18p*-LUC) vs. reporters only, (c) co-infiltrated effectors (BnaA6.RGA+BnaA10.ABF2, or BnaC7.RGA+BnaA10.ABF2) with reporters vs. co-infiltrated effectors (BnaA10.ABF2) with reports, and (d) co-infiltrated effectors (BnaA6.RGA+BnaA10.ABF2, or BnaC7.RGA+BnaA10.ABF2) with reporters vs. co-infiltrated effectors (BnaA6.RGA-ΔGRAS+BnaA10.ABF2, BnaC7.RGA-ΔGRAS+BnaA10.ABF2) with reporters at p < 0.05 (two sided Student’s t test).

**
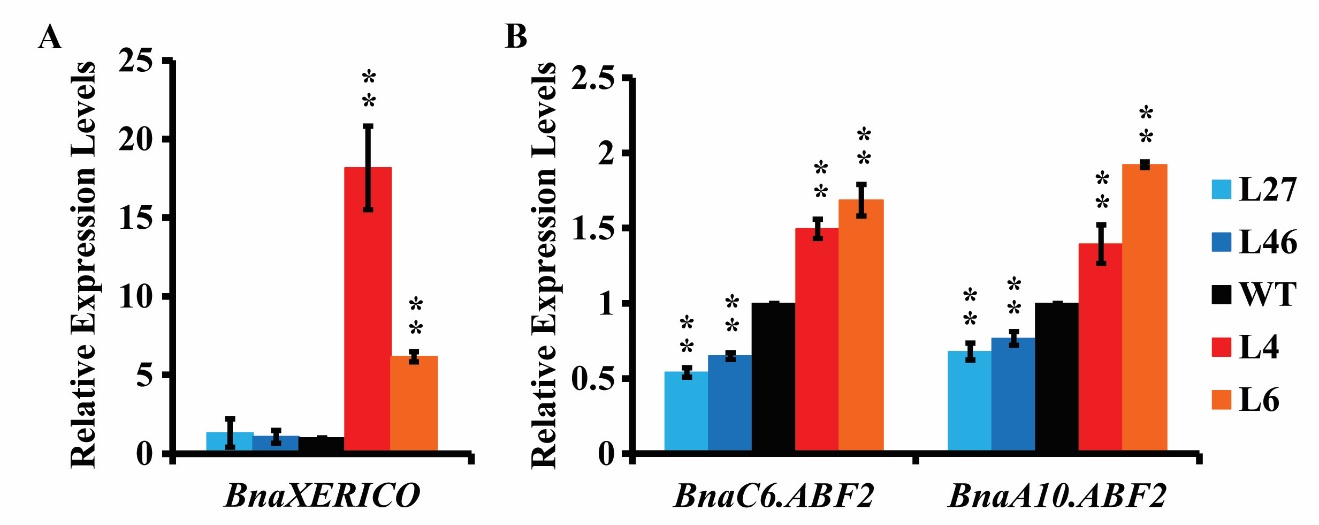
**

**Figure S10. Expression of *BnaXERICO*, *BnaC6.ABF2*, and *BnaA10.ABF2* in *bnaa6.rga-D* and *bnarga* lines.**

(A). Bar graph showing expression of *BnaXERICO* in different mutant lines. (B). Bar graph showing expression of *BnaC6.ABF2* and *BnaA10.ABF2* in different mutant lines. The expressions level of each gene of WT was set as 1. *BnaGAPDH* was used as the internal control. Data are means ± SD obtained from three biological replicates. Asterisks show that the values are significantly different between the WT and mutants at the same development stage. The data were analyzed by Duncan's multiple range tests in the ANOVA program of SPSS (*, P < 0.05; **, P < 0.01). In (A) and (B), L4 and L6: two individual *bnaa6.rga-D* lines; L27 and L46: two individual *bnarga* lines; WT: *Westar*.

**
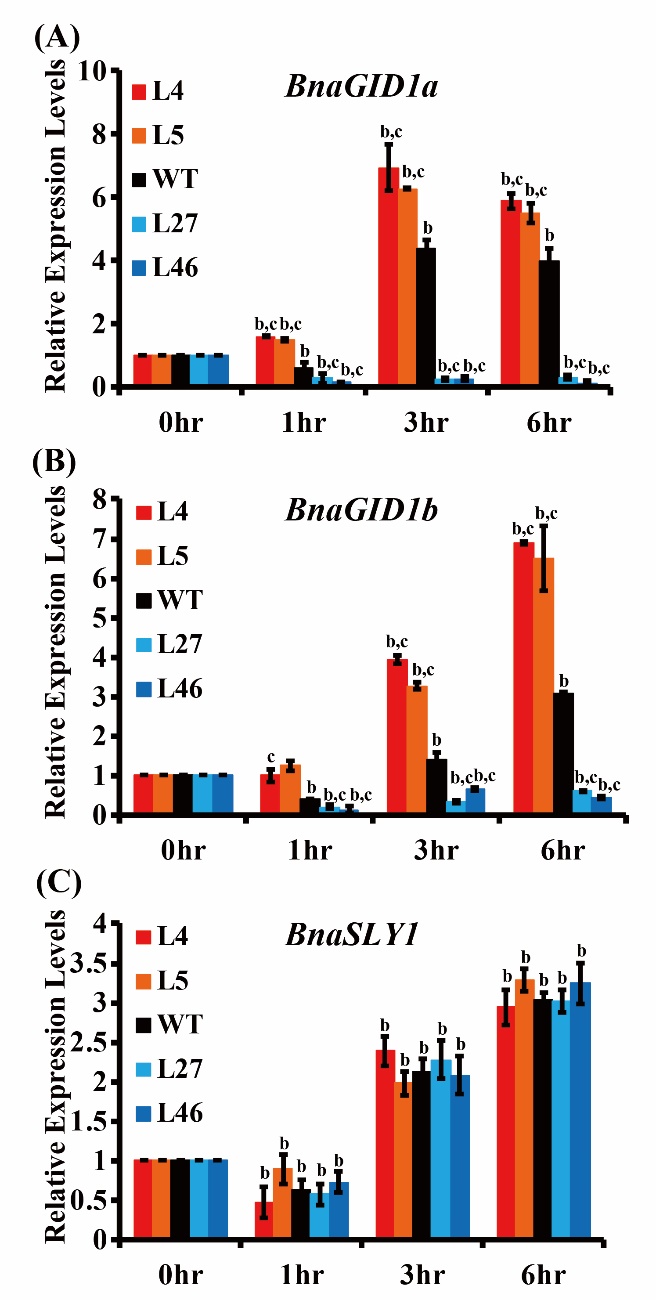
**

**Figure S11. Expression of *BnaGID1a/c* and *BnaSLY1* genes in response to drought treatment.**

(A) to (C) Bar graph showing expression levels of *BnaGID1a* (A), *BnaGID1c* (B) and *BnaSLY1/2* (C) were examined after drought treatment by qRT-PCR. *BnaGAPDH* was used as the internal control. Data are means ± SD obtained from three biological replicates. In (A)-(C), letters indicate statistically significant differences between b: drought treatment vs. control WT, and c: drought treatment of mutants vs. drought treatment of WT at p < 0.05 (Duncan's multiple range tests). In (A)-(C), L4 and L6: two individual *bnaa6.rga-D* lines; L27 and L46: two individual *bnarga* lines; WT: *Westar*.

**Table S1. The list of primers used for making constructs.**

| **Construct** | **Primer name** | **Primer sequence (5'-3')** |
| --- | --- | --- |
| 35S::BnaA9.RGA-YFP-N | BnaA9/C9.RGA-BamHI-F | CGGGATCCATGAAGAGAGATCTCCATCAG |
|  | BnaA9.RGA-XbaI-R | GCTCTAGAAGCACCCATTATTCTCCTC |
| 35S::BnaC9.RGA-YFP-N | BnaA9/C9.RGA-BamHI-F | CGGGATCCATGAAGAGAGATCTCCATCAG |
|  | BnaC9.RGA-XbaI-R | GCTCTAGAAACACCCATTATTCTCCTCC |
| 35S::BnaC7.RGA-YFP-N | BnaC7.RGA-BamHI-F | CGGGATCCATGAAGAGAGATCTTCATCAGTTCCAAGGTCCC |
|  | BnaC7.RGA-XbaI-R | GCTCTAGAGTGCACCGCCGAGAGCTTC |
| 35S::BnaA6.RGA-YFP-N | BnaA6.RGA-BamHI-F | CGGGATCCATGAAGAGGGATCTTCATCAGTTCCAAGG |
|  | BnaA6.RGA-XbaI-R | GCTCTAGAGTGCACCGCCGAGAGCTTC |
| 35S::BnaA10.ABF2-YFP-C | BnaA10.ABF2-XbaI-F | GCTCTAGAATGAAATGCATGATTTTGATGACAAG |
|  | BnaA10.ABF2-BamHI | CGGGATCCCCAAGGTCCTGACTCTGTCCTC |
| 35S::BnaC7.RGA-GFP | BnaC7.RGA-SalI-F | GATACATATGCCCGTCGACCCAAGGTCCTGACTCTGTCCTC |
|  | BnaC7.RGA-BamHI-R | GCTCACCATGAATTCGGATCCGTGCACCGCCGAGAGCTTC |
| 35S::BnaA6.RGA-GFP | BnaA6.RGA-SalI-F | GATACATATGCCCGTCGACATGAAGAGGGATCTTCATCAGTTCCAAGG |
|  | BnaA6.RGA-BamHI-R | GCTCACCATGAATTCGGATCCGTGCACCGCCGAGAGCTTC |
| 35S::BnaA10.ABF2-GFP | BnaA10.ABF2-SalI-F | GATACATATGCCCGTCGACATGAAATGCATGATTTTGATGACAAG |
|  | BnaA10.ABF2-BamHI-R | GCTCACCATGAATTCGGATCCCCAAGGTCCTGACTCTGTCCTC |
| pGBKT7-BnaA10.ABF2 | BnaA10.ABF2-NdeI-F | GGAATTCCATATGATGAAATGCATGATTTTGATGACAAG |
|  | BnaA10.ABF2-BamHI-R | CGGGATCCCCAAGGTCCTGACTCTGTCCTC |
| BnaC9.RAB18p::LUC | C9-RAB18p-KpnI-F | GGGGTACCATTTCACATATAATTCTTACTCGC |
|  | C9-RAB18p-XhoI-R | CCGCTCGAGCTTCTTTTTCTTCTAAGCAAAGACT |
| 35S::BnaA6.RGA-DELLA-YFP-N | YN-RGA.A6-D-F | CGGGATCCCCACCCAATGCTCGATTG |
|  | YN-RGA.A6-D-R | GCTCTAGACGTTAGATCCGGTCGTTGCA |
| 35S::BnaC7.RGA-DELLA-YFP-N | YN-RGA.C7-D-F | CGGGATCCCCACCCAATGCTCGATTGCTTA |
|  | YN-RGA.C7-D-R | GCTCTAGATAATCGCCGCTAGACCCG |
| 35S::BnaA6.RGA-DELLA-GFP | RGA.A6-D-F | GGAATTCCATATGATGAAGAGGGATCTTCATCA |
|  | RGA.A6-D-R | GACTAGTATTAGATCTGCGACAAACC |
| 35S::BnaC7.RGA-DELLA-GFP | RGA.C7-D-F | GGAATTCCATATGATGAAGAGGGATCTTCATCA |
|  | RGA.C7-D-R | GACTAGTATTAGATCCCCTGCAAATCG |

**Table S2. The list of primers used for qRT-PCR.**

| **Gene name** | **Gene ID** | **Direction** | **Primer sequence (5'-3')** | **Product Size (bp)** |
| --- | --- | --- | --- | --- |
| *BnaA10.ABF2* | BnaA10g28780D | Forward | TCTCAGAGTCAGAGGCAGCA | 211 |
|  |  | Reverse | ACACTGGCGTAACAGGAGACA |  |
| *BnaC9.RAB18* | BnaC09g08130D | Forward | TTGGGAGGAATGCTTCACCG | 249 |
|  |  | Reverse | ACCACCGGGAAGCTTTTCCTT |  |
| *BnaRD29A* | BnaC03g15510D | Forward | CCAAGGTTACTGATCCCACTCA | 308 |
|  |  | Reverse | CGGTGAGAACTGATCATGAGTCCC |  |
| *BnaRD29B* | BnaA03g12660D | Forward | CCAAGGTTACTGATCCCACTCA | 256 |
|  |  | Reverse | GAGAACTGATCATGAGTCCCCGT |  |
| *BnaA9.RGA* | BnaA09g18700D | Forward | TGATAACAGTCAGAGCACCAAG | 164 |
|  |  | Reverse | AATCGTTGTTCTGGATTGCTTC |  |
| *BnaC7.RGA* | BnaC07g20900D | Forward | GCTGCATTACTATTCGACCTTG | 282 |
|  |  | Reverse | CCATTATTCTCCTCCACACGAT |  |
| *BnaA6.RGA* | BnaA06g34810D | Forward | CAACGACCGGATCTAACGCTTTG | 244 |
|  |  | Reverse | GACTCGGTCACGGTTGTAAC |  |
| *BnaC9.RGA* | BnaC09g52270D | Forward | ATGGTTTATCTAATCTCGCGACG | 310 |
|  |  | Reverse | GTTGTTACCGTTGTTCCTGTAA |  |
| *BnaC6.ABF2* | BnaC06g00420D | Forward | ACAAGGTTCGATATACTCGT | 155 |
|  |  | Reverse | GAATAACACCGCTAGTTGAAGC |  |
| *BnaABA1* | BnaA09g07610D | Forward | ATGCGTTTCATGAGGAGCCA | 190 |
|  |  | Reverse | GTAACACGCCCTTTACCCCA |  |
| *BnaABA2* | BnaC06g41140D | Forward | ACAACGCAGGAGTAAGCGAA | 276 |
|  |  | Reverse | GTATCCCGTGCTGTCCTAGC |  |
| *BnaGID1a* | BnaA05g32040D | Forward | AGACCAAGAGGGAGGACCT | 273 |
|  |  | Reverse | CTAAGCCAACCCCTCGTGT |  |
| *BnaGID1c* | BnaA09g20650D | Forward | GCCGAGCTTCACCGATCTT | 247 |
|  |  | Reverse | AGCTCTCGAGTTGACCCAG |  |
| *BnaSLY1* | BnaA01g13690D | Forward | TCCCATCTCCCTCGATTCA | 182 |
|  |  | Reverse | GATTCTCGTCTAAGTTGGCG |  |
| *BnaGAPDH* | BnaC05g12400D | Forward | GCGGCGAGATCTCTCTGTTC | 172 |
|  |  | Reverse | CTGGACGAGGTCAGGTTGTG |  |
